# Preliminary Study on Novel Expedient Synthesis of 5-Azaisocoumarins by Transition Metal-Catalyzed Cycloisomerization

**DOI:** 10.3389/fchem.2020.00772

**Published:** 2020-09-04

**Authors:** Jeong A Yoon, Changjin Lim, Young Taek Han

**Affiliations:** ^1^College of Pharmacy, Dankook University, Cheonan-si, South Korea; ^2^School of Pharmacy, Jeonbuk National University, Jeonju-si, South Korea

**Keywords:** 5-azaisocoumarin, cycloisomerization, synthetic method, AgOTf, *N*-pyranonyl propargylamine, Ag(I)-catalysis

## Abstract

A preliminary study to develop a novel synthetic method for 3-aryl-5-azaisocoumarins was performed herein. The cycloisomerization of *N*-pyranonyl propargylamines in the AgOTf-catalyzed system efficiently afforded the desired 3-aryl-5-azaisocoumarins in a highly regioselective manner. This unprecedented method is expected as an expedient alternative synthetic route to 5-azaisocoumarins because the regioselectivity problem is circumvented, and it is easier to introduce substituents on the pyridine ring compared to previously reported intramolecular lactonization approaches.

## Introduction

Isocoumarins, including 3,4-dihydroisocoumarin, are naturally abundant flavonoid scaffolds, that exhibit diverse pharmacological activities (Saeed, 2016; Saddiqa et al., 2017). In particular, 3-aryl isocoumarins possess unique and potent biological functions including antifungal (Saeed, 2003), anti-diabetic (Kim et al., 2017), immunomodulatory (Matsuda et al., 1998), antiallergic (Yoshikawa et al., 1992, 1994, 1996), and antimicrobial (Yoshikawa et al., 1992, 1994, 1996) activities, attracting the attention of synthetic and medicinal chemists. In medicinal chemistry, pyridine is considered to be the most efficient and popular bioisosteres with a phenyl moiety to improve biological activities and PK/PD profiles (Foye, 2008; Gaikwad et al., 2012). In this regard, azaisocoumarins, including 3-aryl-5-azaisocoumarin, are potential privileged scaffolds for developing drug candidates to treat a diverse range of diseases. Hence, there has been significant interest in the development of an efficient and novel synthetic route to 3-aryl-5-azaisocoumarin.

**Figure 1:**
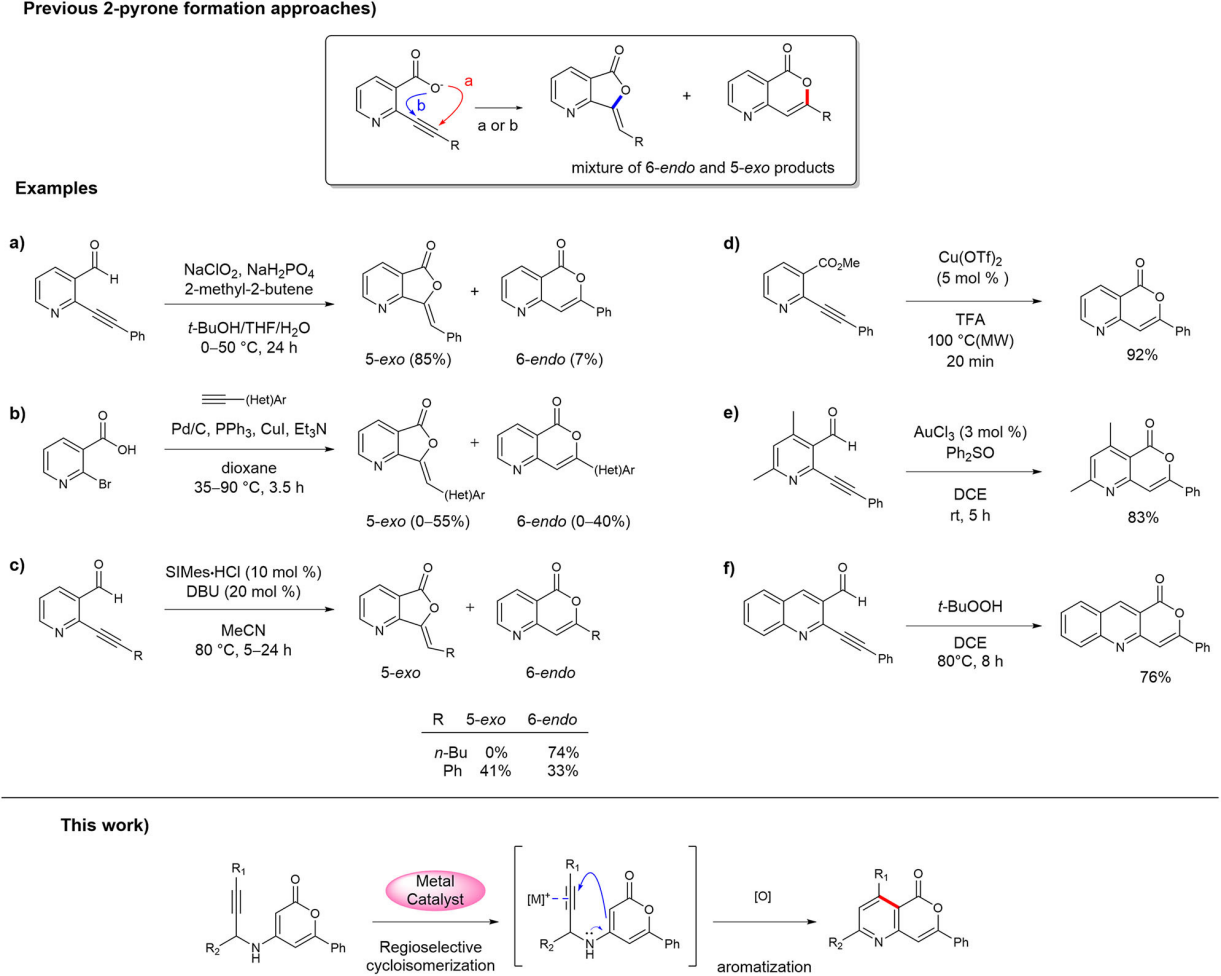
Approaches for the synthesis of 5-azaisocoumarins. **(a)** Li's synthesis of 5-azaisocoumarins; **(b)** Queiroz's synthesis of 5-azaisocoumarins; **(c)** Youn's synthesis of 5-azaisocoumarins; **(d)** Bihel's synthesis of 5-azaisocoumarins; **(e)** Sarkar's synthesis of 5-azaisocoumarins; **(f)** Singh's synthesis of 5-azaisocoumarins.

Despite the continual development of synthetic strategies for 3-aryl-isocoumarin analogs because of their interesting biological activities (Pal et al., 2011; Saddiqa et al., 2017), only a few methods and examples have been reported for the synthesis of 3-aryl-5-azaisocoumarin (Hellal et al., 2008; Li et al., 2010; Begouin and Maria-João, 2011; Panda et al., 2011; Park et al., 2011; Singh et al., 2018). To date, the synthesis of 3-aryl-5-azaisocoumarins has mostly been accomplished in the same manner as that of 3-aryl-isocoumarins, i.e., via construction of the 2-pyrone moiety in the final step via the intramolecular lactonization of 2-alkylnyl nicotinate derivatives. Because lactonization between the activated alkyne and *ortho*-carboxylate can occur by the 6-*endo*-dig or 5-*exo*-dig cyclization sequence (pathways a and b, respectively, in Figure 1), selectivity against methylenefuranone skeleton is a major challenge in these 2-pyrone formation routes. For instance, Li et al. reported the synthesis of 3-aryl-5-azaisocoumarin the Pinnick-type oxidative lactonization of 2-(phenylethynyl)-nicotinaldehyde in 2010 (Figure 1a) (Li et al., 2010). The transition metal-mediated intramolecular lactonizations of 2-alkynyl nicotinates or 2-alkynyl nicotinaldehyde are popular approaches to obtain 5-azaisocoumarins by construction of the 2-pyrone moiety. Queiroz et al. attempted to make the 2-pyrone moiety of 3-aryl-5-azaisocoumarin via the one-pot Sonogashira coupling/transition metal catalyzed alkyne activation cascade (Figure 1b) (Begouin and Maria-João, 2011). These two methods produced 5-*exo*-cyclized furanone as a major product. Youn et al. adopted NHC-catalyzed alkyne activation for the synthesis of 5-azaisocoumarins, which selectively afforded a 6-*endo* or 5-*exo* cyclized product with appropriate reaction conditions (Figure 1c) (Park et al., 2011). 3-Butyl-5-azaisocoumarin was selectively synthesized via 6-*endo* cyclization mode from the corresponding precursor. However, for the arylethynyl precursor, the major product was the 5-*exo*-cyclized lactone instead of 3-aryl-5-azaisocoumarin. Several successful examples of 3-aryl-5-azaisocoumarin synthesis via selective formation of the 2-pyrone moiety have been reported. The Bihel (Figure 1d) (Hellal et al., 2008) and Sarkar groups (Figure 1e) (Panda et al., 2011) reported 3-aryl-5-azaisocoumarin synthesis by transition metal-catalyzed 6-*endo*-lactonization from ethynylnicotinate or ethynylnicotinaldehyde precursors, respectively. In addition, selective 6-*endo*-lactonization toward pyrano[4,3-*b*]quinolin-1-one, a phenyl-fused azaisocoumarin, was accomplished under metal/additive-free domino reaction conditions (Figure 1f) (Singh et al., 2018). In addition to the selectivity problem, previous 2-pyrone formation approaches are inefficient for the installation of substituents on pyridine moiety of 5-azaisocoumarins.

Recently, the metal-catalyzed 6-*endo*-dig cycloisomerization of *N*-propargyl enaminone derivatives has been widely used for the synthesis of pyridine-fused complex heterocycles, including azaanthraquinone, and steroidal pyridine (Abbiati et al., 2003; Yan et al., 2007; Jiang et al., 2010; Fei et al., 2011). In addition, intensive efforts to establish efficient synthetic methods for pyridine-fused coumarins and natural products using the metal-catalyzed cycloisomerization of *N*-propargyl enaminone intermediates have been reported (Ahn et al., 2019; Yoon and Han, 2019). In these studies, the pyridine moiety was constructed in a highly 6-*endo*-selective manner, which can be explained by σ-bond formation between the nucleophilic enolizable α-position and exo-position of the electrophilic alkyne-metal complex and subsequent spontaneous oxidation to form a stable aromatic pyridine ring (Abbiati et al., 2003; Yan et al., 2007; Cacchi et al., 2008; Jiang et al., 2010; Fei et al., 2011; Mikušek et al., 2016; Lyubov'N et al., 2018; Ahn et al., 2019). It was anticipated that construction of the pyridine moiety via cycloisomerization of *N*-propargylamine intermediates in the last-stage would be an efficient strategy for the synthesis of 3-aryl-5-azaisocoumarins. As part of our effort to develop a novel synthetic approach toward privileged 3-aryl-5-azaisocoumarin scaffolds, we investigated the metal-catalyzed cycloisomerization of *N*-pyranonyl propargylamines.

Herein, we describe a concise synthesis of 3-aryl-5-azaisocoumarins via AgOTf-catalyzed cycloisomerization, which to the best of our knowledge, has not been reported to date.

## Materials and Methods

### General Information

Unless noted otherwise, all starting materials and reagents were obtained commercially and were used without further purification. All solvents utilized for routine product isolation and chromatography were of reagent grade and glass-distilled, and reaction flasks were dried at 100°C before use. Flash column chromatography was performed using silica gel 60 (230–400 mesh, Merck, Kenilworth, NJ, USA) with the indicated solvents. Thin-layer chromatography (TLC) was performed using 0.25-mm silica gel plates (Merck, Kenilworth, NJ, USA). Mass spectra were obtained using an Agilent 6530 Q-TOF (Santa Clara, CA, USA) instrument. Infrared spectra were recorded on a JASCO FT-IR-4200 spectrometer (Tokyo, Japan). ^1^H and ^13^C spectra were recorded on a Brucker Analytik ADVANCE digital 500 (500 MHz) (Billerica, MA, USA) and BRUKER AVANCE-800 (Billerica, MA, USA). Chemical shifts are expressed in parts per million (ppm, δ) downfield from tetramethylsilane and are referenced to the deuterated solvent. ^1^HNMR data are reported in the order of chemical shift, multiplicity (s, singlet; d, doublet; t, triplet; q, quartet; m, multiplet; bs, broad singlet; and/or multiple resonances), number of protons, and coupling constant in hertz (Hz).

#### 2-Oxo-6-phenyl-2H-pyran-4-yl 4-methylbenzenesulfonate (2)

To a stirred solution of **1** (500 mg, 2.66 mmol) in CH_2_Cl_2_ (20 mL) were added tosyl chloride (507 mg, 2.66 mmol) and Et_3_N (1.11 mL, 7.98 mmol) at ambient temperature. After stirring for 3 h, the reaction mixture was quenched with water and extracted with CH_2_Cl_2_. The combined organic layer was washed with brine, dried over MgSO_4_, and concentrated *in vacuo*. The residue was purified by flash column chromatography (EtOAc/*n*-Hexane = 1: 5) to give 838 mg (92%) of **2** as a yellow solid.

m.p.: 128–130°C; R_f_ = 0.67 (EtOAc/*n*-Hexane = 1: 1); ^1^H-NMR (500 MHz, CDCl_3_) δ 7.86 (d, 2H, *J* = 8.3 Hz, Ar-H), 7.78 (d, 2H, *J* = 6.9 Hz, Ar-H), 7.51–7.45 (m, 3H, Ar-H), 7.40 (d, 2H, *J* = 8.0 Hz, Ar-H), 6.61 (d, 1H*, J* = 1.8 Hz, vinyl H), 5.91 (d, 1H, *J* = 1.8 Hz, cyclic-CH), 2.48 (s, 3H, CH_3_); ^13^C-NMR (125 MHz, CDCl_3_) δ 162.4, 162.3, 162.2, 146.9, 131.9, 131.8, 130.5, 130.5, 129.2, 128.6, 126.0, 101.7, 98.3, 22.0; IR (thin film, neat) ν_max_ 1,736, 1,631, 1,556, 1,496, 1,386, 1,198, 1,139, 1,091, 916, 748 cm^−1^; LR-MS (ESI+) *m/z* 343 (M + H^+^); HR-MS (ESI+) calcd for C_18_H_15_O_5_S (M + H^+^) 343.0635; found 343.0631.

### General Procedure I for Nucleophilic Aromatic Substitution

To a stirred solution of **2** (1 equiv.) and *N,N*-diisopropylethylamine (3 equiv.) in MeCN (0.1 M solution) was added propargylamine hydrochloride (1.5 equiv.) at ambient temperature. After stirring for 14 h at 80°C, the reaction mixture was quenched with 1*N* HCl and extracted with EtOAc. The combined organic layer was washed with brine, dried over MgSO_4_, and concentrated *in vacuo*. The residue was purified by flash column chromatography (EtOAc/*n*-Hexane = 1: 1) to give title compounds (**3a**–**3i**).

#### 6-Phenyl-4-(prop-2-yn-1-ylamino)-3,4-dihydro-2H-pyran-2-one (3a)

Yellow solid of **3a** (1.13 g, 69%) was obtained via General Procedure I from 2.50 g of **2**.

m.p.: 169–171°C; R_f_ = 0.29 (EtOAc/*n*-Hexane = 1: 1); ^1^H-NMR (500 MHz, DMSO-d_6_) δ 7.72 (s, 3H, Ar-H), 7.54–7.51 (m, 2H, Ar-H), 6.55 (s, 1H, vinyl H), 5.03 (d, 1H, *J* = 1.3 Hz, vinyl H), 4.01 (s, 2H, CH_2_), 3.30 (t, 1H, *J* = 2.3 Hz, CH); ^13^C-NMR (125 MHz, DMSO-d_6_) δ 162.5, 157.8, 157.1, 131.6, 130.6, 129.1, 125.1, 97.0, 80.7, 79.5, 74.6, 31.3; IR (thin film, neat) ν_max_ 3,289, 3,089, 1,666, 1,549, 1,298, 1,198, 1,063, 847, 767, 691 cm^−1^; LR-MS (ESI+) *m/z* 226 (M + H^+^); HR-MS (ESI+) calcd for C_14_H_12_NO_2_ (M + H^+^) 226.0863; found 226.0856.

#### 4-(But-2-yn-1-ylamino)-6-phenyl-3,4-dihydro-2H-pyran-2-one (3b)

Yellow solid of **3b** (25 mg, 72%) was obtained via General Procedure I from 50 mg of **2**.

m.p.: 181–183°C; R_f_ = 0.38 (EtOAc/*n*-Hexane = 1: 1); ^1^H-NMR (500 MHz, DMSO-d_6_) δ 7.72 (s, 2H, Ar-H), 7.68 (t, 1H, *J* = 5.3 Hz, NH), 7.54–7.49 (m, 3H, Ar-H), 6.54 (s, 1H, vinyl H), 4.99 (s, 1H, vinyl H), 3.94 (s, 2H, CH_2_), 1.81 (t, 3H, *J* = 2.1 Hz, CH_3_); ^13^C-NMR (125 MHz, DMSO-d_6_) δ 162.5, 157.7, 157.2, 131.6, 130.5, 129.1, 125.1, 97.0, 80.4, 79.6, 74.6, 31.6, 3.0; IR (thin film, neat) ν_max_ 3,269, 3,090, 1,658, 1,547, 1,298, 1,194, 1,063, 992, 850, 768, 692 cm^−1^; LR-MS (ESI+) *m/z* 240 (M + H^+^); HR-MS (ESI+) calcd for C_15_H_14_NO_2_ (M + H^+^) 240.1019; found 240.1018.

#### 4-(But-3-yn-2-ylamino)-6-phenyl-3,4-dihydro-2H-pyran-2-one (3c)

Yellow solid of **3c** (17 mg, 48%) was obtained via General Procedure I from 50 mg of **2**.

m.p.: 190–191°C; R_f_ = 0.30 (EtOAc/*n*-Hexane = 1: 1); ^1^H-NMR (500 MHz, DMSO-d_6_) δ 7.72 (s, 2H, Ar-H), 7.66 (d, 1H, *J* = 6.9 Hz, NH), 7.54–7.50 (m, 3H, Ar-H), 6.53 (s, 1H, vinyl H), 5.06 (d, 1H, *J* = 1.5 Hz, vinyl H), 4.37 (s, 1H, CH), 3.32 (d, 1H, *J* = 2.1 Hz, CH), 1.43 (d, 3H*, J* = 6.8 Hz, CH_3_); ^13^C-NMR (125 MHz, DMSO-d_6_) δ 162.5, 156.9, 156.4, 131.6, 130.5, 129.1, 126.9, 125.1, 97.0, 83.8, 81.0, 73.5, 20.9; IR (thin film, neat) ν_max_ 3,283, 3,066, 1,667, 1,550, 1,298, 1,198, 1,176, 1,063, 847, 758, 691 cm^−1^; LR-MS (ESI+) *m/z* 240 (M + H^+^); HR-MS (ESI+) calcd for C_15_H_14_NO_2_ (M + H^+^) 240.1019; found 240.1029.

#### 6-Phenyl-4-((3-phenylprop-2-yn-1-yl)amino)-3,4-dihydro-2H-pyran-2-one (3d)

Yellow solid of **3d** (24 mg, 52%) was obtained via General Procedure I from 50 mg of **2**.

m.p.: 194–196°C; R_f_ = 0.24 (EtOAc/*n*-Hexane = 1: 1); ^1^H-NMR (500 MHz, DMSO-d_6_), δ 7.83 (t, 1H, *J* = 5.3 Hz, Ar-H), 7.74 (s, 1H, Ar-H), 7.54–7.51 (m, 3H, Ar-H), 7.45–7.38 (m, 5H, Ar-H), 6.59 (d, 1H, *J* = 6.6 Hz, vinyl H), 5.12 (d, 1H, *J* = 1.3 Hz, vinyl H), 4.28 (s, 2H, CH_2_); ^13^C-NMR (125 MHz, DMSO-d_6_) δ 162.5, 157.9, 157.2, 131.6, 131.4, 130.6, 129.1, 128.8, 128.7, 125.1, 121.9, 97.1, 85.4, 83.1, 80.7, 32.1; IR (thin film, neat) ν_max_ 3,271, 3,089, 1,663, 1,544, 1,451, 1,293, 1,199, 1,072, 849, 768, 691 cm^−1^; LR-MS (ESI+) *m/z* 324 (M + Na^+^); HR-MS (ESI+) calcd for C_20_H_15_NNaO_2_ (M + Na^+^) 324.0995; found 324.0990.

#### 6-phenyl-4-((3-(o-tolyl)prop-2-yn-1-yl)amino)-2H-pyran-2-one (3e)

Yellow solid of **3e** (69 mg, 75%) was obtained via General Procedure I from 100 mg of **2**.

m.p.: 163–164°C; R_f_ = 0.40 (EtOAc/*n*-Hexane = 1: 1); ^1^H-NMR (800 MHz, DMSO-d_6_) δ 7.84 (s, 1H, NH), 7.74 (s, 2H, Ar-H), 7.55–7.48 (m, 3H, Ar-H), 7.38 (d, *J* = 7.6 Hz, 1H, Ar-H), 7.30–7.23 (m, 2H, Ar-H), 7.22 – 7.12 (m, 1H, Ar-H), 6.59 (s, 1H, vinyl H), 5.15 (d, *J* = 1.2 Hz, 1H, vinyl H), 4.31 (s, 2H, CH_2_), 2.36 (s, 3H, CH_3_); ^13^C-NMR (200 MHz, DMSO-d_6_) δ 162.5, 157.8, 157.1, 139.7, 131.6, 131.6, 130.6, 129.6, 129.1, 128.7, 125.9, 125.1, 121.8, 97.1, 89.2, 82.0, 80.9, 32.2, 20.2; IR (thin film, neat) ν_max_ 3,263, 3,072, 1,664, 1,544, 1,289, 1,182, 1,027, 759, 689 cm^−1^; LR-MS (ESI+) *m/z* 316 (M + H^+^); HR-MS (ESI+) calcd for C_21_H_18_NO_2_ (M + H^+^) 316.1332; found 316.1333.

#### 4-(hex-5-en-2-yn-1-ylamino)-6-phenyl-2H-pyran-2-one (3g)

Yellow solid of **3g** (53 mg, 68%) was obtained via General Procedure I from 100 mg of **2**.

m.p.: 119–120°C; R_f_ = 0.39 (EtOAc/*n*-Hexane = 1: 1); ^1^H-NMR (800 MHz, DMSO-d_6_) δ 7.82–7.65 (m, 3H, Ar-H & NH), 7.54–7.48 (m, 3H, Ar-H), 6.55 (s, 1H, vinyl H), 5.87–5.69 (ddt, *J* = 17.0, 10.1, 5.2 Hz, 1H, vinyl H), 5.30 (dd, *J* = 17.0, 1.7 Hz, 1H, vinyl H), 5.10 (d, *J* = 9.7 Hz, 1H, vinyl H), 5.03 (d, *J* = 1.5 Hz, 1H, vinyl H), 4.02 (s, 2H, CH_2_), 3.05–3.01 (m, 2H, CH_2_); ^13^C-NMR (200 MHz, DMSO-d_6_) δ 162.6, 157.7, 157.1, 132.8, 131.6, 130.6, 129.1, 125.1, 116.0, 97.0, 80.5, 80.3, 78.1, 31.7, 22.3; IR (thin film, neat) ν_max_ 3,272, 3,092, 1,664, 1,548, 1,297, 1,182, 1,038, 918, 767, 691 cm^−1^; LR-MS (ESI+) *m/z* 266 (M + H^+^); HR-MS (ESI+) calcd for C_17_H_16_NO_2_ (M + H^+^) 266.1176; found 266.1169.

#### 4-((3-(cyclohex-2-en-1-yl)prop-2-yn-1-yl)amino)-6-phenyl-2H-pyran-2-one (3h)

Yellow oil of **3h** (39 mg, 44%) was obtained via General Procedure I from 100 mg of **2**.

R_f_ = 0.35 (EtOAc/*n*-Hexane = 1: 1); ^1^H-NMR (800 MHz, DMSO-d_6_) δ 7.72 (s, 2H, Ar-H), 7.66 (s, 1H, NH), 7.54–7.48 (m, 3H, Ar-H), 6.55 (s, 1H, vinyl H), 5.72–5.68 (m, 1H, vinyl H), 5.58–5.55 (m, 1H, vinyl H), 5.00 (s, 1H, vinyl H), 3.98 (s, 2H, CH_2_), 3.18–3.11 (m, 1H, CH), 1.98–1.91 (m, 2H, 2 × CH_2_), 1.85–1.79 (m, 1H, 1/2 × CH_2_), 1.73–1.67 (m, 1H, 1/2 × CH_2_), 1.60–1.55 (m, 1H, 1/2 × CH_2_), 1.54–1.48 (m, 1H, 1/2 × CH_2_); ^13^C-NMR (200 MHz, DMSO-d_6_) δ 162.5, 157.7, 157.1, 131.6, 130.5, 129.1, 127.8, 126.8, 125.1, 97.1, 86.4, 80.4, 75.3, 31.7, 28.9, 26.6, 24.1, 20.0; IR (thin film, neat) ν_max_ 3,117, 2,887, 1,670, 1,564, 1,337, 1,245, 767, 688 cm^−1^; LR-MS (ESI+) *m/z* 306 (M + H^+^); HR-MS (ESI+) calcd for C_20_H_20_NO_2_ (M + H^+^) 306.1489; found 306.1486.

#### 6-phenyl-4-((3-(triisopropylsilyl)prop-2-yn-1-yl)amino)-2H-pyran-2-one (3i)

Yellow solid of **3i** (80 mg, 72%) was obtained via General Procedure I from 100 mg of **2**.

m.p.: 175–176°C; R_f_ = 0.60 (EtOAc/*n*-Hexane = 1: 1); ^1^H-NMR (800 MHz, DMSO-d_6_) δ 7.74 (s, 2H, Ar-H), 7.68 (s, 1H, NH), 7.54–7.47 (m, 3H, Ar-H), 6.57 (s, 1H, vinyl H), 5.07 (d, *J* = 1.9 Hz, 1H, vinyl H), 4.09 (s, 2H, CH_2_), 1.05–0.98 (m, 21H, TIPS-H); ^13^C-NMR (200 MHz, DMSO-d_6_) δ 162.4, 157.7, 157.0, 131.6, 130.5, 129.1, 125.1, 103.6, 97.0, 83.6, 81.1, 32.3, 18.4, 10.6; IR (thin film, neat) ν_max_ 3,272, 3,075, 1,670, 1,549, 1,245, 1,182, 1,038, 883, 767, 677 cm^−1^; LR-MS (ESI+) *m/z* 382 (M + H^+^); HR-MS (ESI+) calcd for C_23_H_32_NO_2_Si (M + H^+^) 382.2197; found 382.2193.

### General Procedure II for AgOTf-Catalyzed Cycloisomerization

To a stirred solution of **3a**–**3f** (1 equiv.) in DMSO (0.06 M) was added AgOTf (0.2 equiv.) at ambient temperature. After stirring for 2–4 h at 120°C, the reaction mixture was cooled to ambient temperature, quenched with water and extracted with EtOAc. The combined organic layer was washed with brine, dried over MgSO_4_, and concentrated *in vacuo*. The residue was purified by flash column chromatography (EtOAc/*n*-Hexane = 1: 3) to give title compounds (**4a**–**4f**).

#### 7-Phenyl-5H-pyrano[4,3-b]pyridin-5-one (4a)

Yellow solid of **4a** (13 mg, 66%) was obtained via General Procedure II from 20 mg of **3a**.

m.p.: 135–136°C; R_f_ = 0.47 (EtOAc/*n*-Hexane = 1: 1); ^1^H-NMR (800 MHz, CDCl_3_) δ 8.94 (dd, 1H, *J* = 1.8, 4.6 Hz, Ar-H), 8.55 (dd, 1H, *J* = 1.4, 7.8 Hz, Ar-H), 7.93–7.91 (m, 2H, Ar-H), 7.51–7.47 (m, 3H, Ar-H), 7.43 (dd, 1H, *J* = 4.6, 7.9 Hz, Ar-H), 7.23 (s, 1H, vinyl H); ^13^C-NMR (125 MHz, CDCl_3_) δ 162.1, 157.6, 156.3, 155.1, 137.9, 131.5, 131.0, 129.2, 125.9, 123.0, 117.2, 103.6; IR (thin film, neat) ν_max_ 3,067, 1,735, 1,633, 1,561, 1,450, 1,216, 1,044, 1,035, 753, 688 cm^−1^; LR-MS (ESI+) *m/z* 224 (M + H^+^); HR-MS (ESI+) calcd for C_14_H_10_NO_2_ (M + H^+^) 224.0706; found 224.0707.

#### 4-Methyl-7-phenyl-5H-pyrano[4,3-b]pyridin-5-one (4b)

Yellow solid of **4b** (16 mg, 65%) was obtained via General Procedure II from 25 mg of **3b**.

m.p.: 113–114°C; R_f_ = 0.58, (EtOAc/*n*-Hexane = 1: 1); ^1^H-NMR (500 MHz, CDCl_3_) δ 8.68 (d, 1H, *J* = 4.9 Hz, Ar-H), 7.93–7.91 (m, 2H, Ar-H), 7.51–7.47 (m, 3H, Ar-H), 7.21–7.20 (m, 2H, Ar-H & vinyl H), 2.86 (s, 3H, CH_3_); ^13^C-NMR (125 MHz, CDCl_3_) δ 161.3, 157.1, 156.2, 154.6, 153.6, 131.4, 130.9, 129.1, 125.8, 125.7, 116.2, 103.8, 22.7; IR (thin film, neat) ν_max_ 3,060, 1,734, 1,637, 1,574, 1,465, 1,228, 1,058, 1,034, 847, 762, 681 cm^−1^; LR-MS (ESI+) *m/z* 238 (M + H^+^); HR-MS (ESI+) calcd for C_15_H_12_NO_2_ (M + H^+^) 238.0863; found 238.0860.

#### 2-Methyl-7-phenyl-5H-pyrano[4,3-b]pyridin-5-one (4c)

Yellow solid of **4c** (11 mg, 54%) was obtained via General Procedure II from 20 mg of **3c**.

m.p.: 129–130°C; R_f_ = 0.53 (EtOAc/*n*-Hexane = 1: 1); ^1^H-NMR (500 MHz, CDCl_3_) δ 8.43 (d, 1H, *J* = 8.1 Hz, Ar-H), 7.93–7.90 (m, 2H, Ar-H), 7.51–7.47 (m, 3H, Ar-H), 7.30 (d, 1H, *J* = 8.1 Hz, Ar-H), 7.22 (s, 1H, vinyl H), 2.72 (s, 3H, CH_3_); ^13^C-NMR (125 MHz, CDCl_3_) δ 166.6, 162.2, 157.6, 154.8, 137.8, 131.6, 130.9, 129.1, 125.8, 123.3, 114.7, 103.6, 25.5; IR (thin film, neat) ν_max_ 3,061, 1,752, 1,630, 1,570, 1,460, 1,241, 1,119, 1,064, 1,030, 762, 687 cm^−1^; LR-MS (ESI+) *m/z* 238 (M + H^+^); HR-MS (ESI+) calcd for C_15_H_12_NO_2_ (M + H^+^) 238.0863; found 238.0857.

#### 4,7-Diphenyl-5H-pyrano[4,3-b]pyridin-5-one (4d)

Yellow solid of **4d** (12 mg, 60%) was obtained via General Procedure II from 20 mg of **3d**.

m.p.: 142–144°C; R_f_ = 0.61 (EtOAc/*n*-Hexane = 1: 1); ^1^H-NMR (500 MHz, CDCl_3_) δ 8.86 (d, 1H, *J* = 4.9 Hz, Ar-H), 7.94–7.92 (m, 2H, Ar-H), 7.50–7.48 (m, 6H, Ar-H), 7.40–7.37 (m, 3H, Ar-H), 7.28 (d, 1H, *J* = 4.2 Hz, vinyl H); ^13^C-NMR (125 MHz, CDCl_3_) δ 160.1, 157.5, 156.4, 154.7, 154.6, 138.5, 131.3, 131.0, 129.1, 128.8, 128.3, 128.2, 125.8, 125.5, 114.3, 103.7; IR (thin film, neat) ν_max_ 3,058, 1,745, 1,637, 1,568, 1,463, 1,222, 1,060, 1,029, 856, 764, 696 cm^−1^; LR-MS (ESI+) *m/z* 300 (M + H^+^); HR-MS (ESI+) calcd for C_20_H_14_NO_2_ (M + H^+^) 300.1019; found 300.1017.

#### 7-phenyl-4-(o-tolyl)-5H-pyrano[4,3-b]pyridin-5-one (4e)

Yellow solid of **4e** (34 mg, 50%) was obtained via General Procedure II from 68 mg of **3e**.

m.p.: 137–139°C; R_f_ = 0.62 (EtOAc/*n*-Hexane = 1: 1); ^1^H-NMR (800 MHz, CDCl_3_) δ 8.90 (s, 1H, Ar-H), 7.91–7.88 (m, 2H, Ar-H), 7.49–7.45 (m, 3H, Ar-H), 7.36–7.32 (m, 2H, Ar-H), 7.30–7.25 (m, 2H, Ar-H), 7.22–7.18 (m, 1H, Ar-H), 7.06 (d, *J* = 6.9 Hz, 1H, vinyl H), 2.09 (s, 3H, CH_3_); ^13^C-NMR (200 MHz, CDCl_3_) δ 159.7, 157.9, 156.0, 154.9, 154.4, 138.5, 134.7, 131.2, 131.1, 123.0, 129.2, 128.6, 127.3, 125.9, 125.8, 125.3, 115.5, 103.3, 20.0; IR (thin film, neat) ν_max_ 3,066, 1,746, 1,636, 1,568, 1,495, 1,223, 1,027, 761, 688 cm^−1^; LR-MS (ESI+) *m/z* 314 (M + H^+^); HR-MS (ESI+) calcd for C_21_H_16_NO_2_ (M + H^+^) 314.1176; found 314.1174.

#### 4-(3-nitrophenyl)-7-phenyl-5H-pyrano[4,3-b]pyridin-5-one (4f)

*N*-pyranonyl propargylamine **3f** was obtained via General Procedure I from 100 mg of **2**. The crude **3f** was used for the next step without further purification. Yellow solid of **4f** (32 mg, 32% for 2 steps) was obtained via General Procedure II from the crude **3f**.

m.p.: 261–262°C; R_f_ = 0.48 (EtOAc/*n*-Hexane = 1: 1); ^1^H-NMR (800 MHz, DMSO-d_6_) δ 9.02 (d, *J* = 4.8 Hz, 1H, Ar-H), 8.38 (t, *J* = 1.9 Hz, 1H, Ar-H), 8.32 (ddd, *J* = 8.3, 2.3, 0.9 Hz, 1H, Ar-H), 8.01 (dd, *J* = 8.1, 1.5 Hz, 2H, Ar-H), 7.95 (ddd, *J* = 7.6, 1.4, 1.1 Hz, 1H, Ar-H), 7.77 (t, *J* = 7.9 Hz, 1H, Ar-H), 7.60 (s, 1H, Ar-H), 7.58–7.53 (m, 3H, Ar-H), 7.47 (d, *J* = 4.8 Hz, 1H, vinyl H); ^13^C-NMR (200 MHz, DMSO-d_6_) δ 159.9, 156.1, 155.8, 155.6, 149.9, 147.3, 140.2, 135.1, 130.9, 130.8, 129.4, 129.2, 125.4, 125.3, 123.5, 123.1, 114.0, 103.9; IR (thin film, neat) ν_max_ 3,115, 1,796, 1,637, 1,542, 1,446, 1,219, 1,038, 856, 759, 676 cm^−1^; LR-MS (ESI+) *m/z* 345 (M + H^+^); HR-MS (ESI+) calcd for C_20_H_13_N_2_O_4_ (M + H^+^) 345.0870; found 345.0867.

## Results and Discussions

**Scheme 1:**
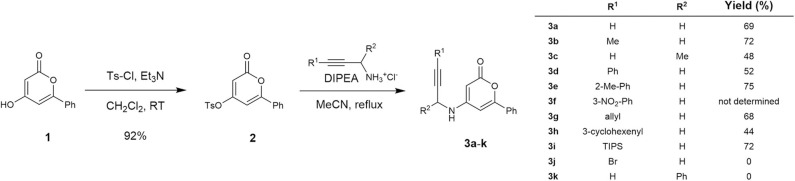
Synthesis of *N*-pyranonyl propargylamines **3a−3k**.

Our research on the development of a novel synthetic route toward 3-aryl-5-azaisocoumarin commenced with the preparation of cycloisomerization precursors. As shown in Scheme 1, *N*-pyranonyl propargylamine precursors (**3a**–**3k**) were prepared according to literature procedures with slight modifications (Dong et al., 2011). The known hydroxy-2-pyrone **1** (Prasad et al., 1995) was treated with tosyl chloride and triethylamine to afford tosylate **2** in 92% yield. Nucleophilic aromatic substitution of pyrone **2** with commercially available hydrochloride salts of propargylamine or diverse known substituted propargylamine (Experimental details: see Supplementary Materials) readily afforded the corresponding *N*-pyranonyl propargylamines **3a**–**3h** in moderate yields (48–74%). However, 3-bromopropargyl- and 1-phenylpropargyl analogs (**3j**–**3k**) were not obtained. The nitrophenyl substituted analog **3i** was used for the next reaction without further purification due to inseparable impurities.

With the cycloisomerization precursors **3a**–**3i** in hand, the cycloisomerization conditions were examined using the simple **3a** as a model substrate (Table 1). Initially, cycloisomerization of **3a** was conducted under thermal rearrangement conditions. When **3a** was heated at 120°C in DMSO for 10 h, the desired 5-azaisocoumarin **4a** was obtained in poor yield (15%, Table 1, entry 1). The reaction was performed at higher temperature (220°C) in triethylene glycol (TEG) (Han, 2017), affording 5-azaisocoumarin **4a** in a slightly higher yield (28%, entry 2). The structure of **4a** was confirmed by comparison of its NMR spectrum with previously reported data (Table S1) (Hellal et al., 2008). Recently, many research groups have continuously reported the efficiency of various transition-metal Lewis acid catalysts for alkyne activation in cycloisomerization reaction (Abbiati et al., 2003; Yan et al., 2007; Cacchi et al., 2008; Saito et al., 2009; Jiang et al., 2010; Fei et al., 2011; Mikušek et al., 2016; Vessally et al., 2016; Nizami and Hua, 2017; Sakthivel et al., 2017; Gianni et al., 2018; Lyubov'N et al., 2018; Mancuso et al., 2018; Ahn et al., 2019). To increase the product yield, cycloisomerization was performed in the presence of various metal catalysts, including Ag(I), Au(I), Cu(I), Cu(II), and In(III). Based on our previous work (Ahn et al., 2019), the cycloisomerization of **3a** was performed with 0.2 equiv. of AgSbF_6_ (entry 3), affording **4a** in a higher yield (38%) than that without the catalyst after heating in DMSO at 120°C for 7 h. Encouraged by this yield improvement with the Ag(I) catalyst, various counter-anions was screened (entries 4–7) given that the alkyne-activating Lewis acidity of metal ions such as Ag(I) depends on the nature of the counter-anion (Yamamoto, 2000; Lu et al., 2017). When Ag_2_O was employed, the yield of **4a** did not notably improve over that obtained with AgSbF_6_ (38%, entry 4). However, the yields increased slightly when AgCl (50%, entry 5) or AgNO_3_ (53%, entry 6) were used. The best result was obtained when the cycloisomerization was performed with AgOTf, furnishing **4a** in 66% yield (entry 7). Moreover, the reaction proceeded to completion much faster (2 h) than with AgSbF_6_. Although regioisomeric byproducts were not isolated as expected, the optimal yield for the cycloisomerization of *N*-pyranonyl propargylamine precursors **3a** was slightly lower than that reported in a previous study on pyridocoumarins (Ahn et al., 2019), likely because of the greater degree of decomposition of the acid-labile pyrone moiety. When the reaction was conducted with AuCl, the yield was reduced compared to that observed in the thermal aza-Claisen rearrangement in TEG (24%, entry 8). Furthermore, the desired cycloisomerization product **4a** was not obtained when using InCl_3_ (entry 9). Cu(I) and Cu(II) catalysts afforded 5-azaisocoumarin **4a** in better yields than with Au(I) or In(III) catalysts (33%−62%; entries 10–14). Although CuI afforded **4a** in the best yield among the copper catalysts tested herein (62%, entry 11), the yield of **4a** and reaction rate did not show any notable improvements over that obtained with AgOTf. Other polar solvents including DMF (entry 15), MeCN (entry 16), and ionic liquid systems (entries 17–18), resulted in lower reaction rates and yields of **4a**. In particular, the use of an ionic liquid hampered the cycloisomerization, contrary to previous reports (Corma et al., 2005; Neatu et al., 2009; Mancuso et al., 2019). The lower yields obtained in ionic liquids was attributed to the inhibition of the interaction between the alkyne and metal catalyst by the highly polar solvent.

**Table 1:** Investigation of reaction conditions for the cycloisomerization of **3a**.

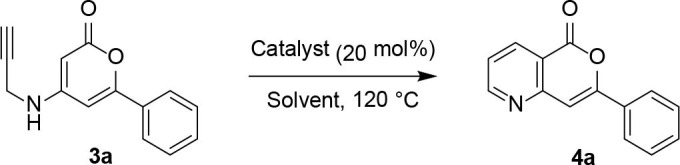				
**Entry**	**Catalyst**	**Solvent**^**a**^	**Time (h)**	**Yield (%)**
1	-	DMSO	10	15
2	-	TEG^b^	3	28
3	AgSbF_6_	DMSO	7	38
4	Ag_2_O	DMSO	5	38
5	AgCl	DMSO	4	50
6	AgNO_3_	DMSO	2	53
7	AgOTf	DMSO	2	66
8	AuCl	DMSO	3	24
9	InCl_3_	DMSO	24	NR^c^
10	Cu_2_O	DMSO	4	61
11	CuI	DMSO	2	62
12	CuCl	DMSO	6	58
13	CuBr	DMSO	4	33
14	Cu(OAc)_2_	DMSO	4	46
15	AgOTf	DMF	5	46
16	AgOTf	MeCN^d^	21	52
17	AgOTf	DMSO/IL^e^	28	25
18	AgOTf	IL^f^	28	NRa^c^

a 0.06 M solution of **3a**;

b *0.05 M solution of **3a** in triethylene glycol at 220–230°C*.

c Starting material was recovered;

d *reflux*.

e,f *IL = Ionic liquid = [EMIm][EtSO_4_], DMSO: [EMIm][EtSO_4_] = 10: 1*.

Next, the scope and limitations of the newly developed Ag(I)-catalyzed 3-aryl-5-azaisocoumarin synthesis were examined (Table 2). We attempted to synthesize the 3-aryl-5-azaisocoumarins from corresponding substituted alkynes (**3b**–**3i**). Methyl (**3b**–**3c**; entry 1–2) and phenyl substituted (**3d**–**3f**; entry 3–5) propargylamines generated the corresponding 3-aryl-5-azaisocoumarins (**4b**–**4f**) without any notable yield decreases. However, cycloisomerization under standard condition of enynylamine precursors (**3g**–**3h**; entry 6–7) resulted in an unidentifiable mixture of byproducts, whereas only substrates were recovered when the reactions were conducted at ambient temperature. These results were attributed to preference of the metal-alkyne complex for reaction with the electron-rich alkene rather than enolizable α-position. Although the cycloisomerization reaction of triisopropylsilyl (TIPS) propargylamine (**3i**) was allowed to proceed for 24 h, only substrate **3i** was recovered in 88% yield (entry 8). This implied that the bulky silyl substituents of alkyne can interfere with formation of the metal-alkyne complex.

**Table 2:** Substrate scope of the AgOTf-catalyzed cycloisomerization toward 3-aryl-5-azaisocoumarin.

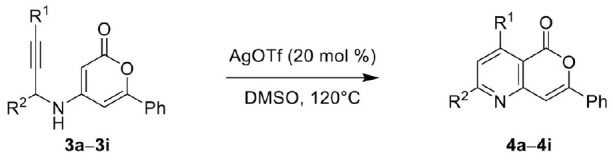				
**Entry**	**Substrate**	**Product**	**Time (h)**	**Yield (%)**
1	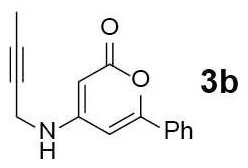	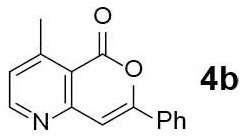	4	65
2	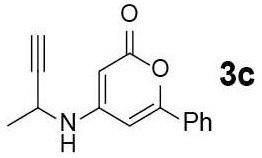	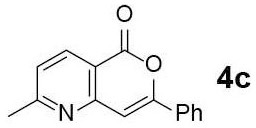	3	54
3	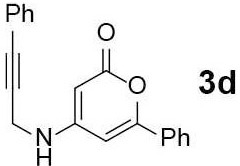	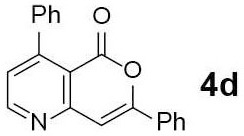	3	60
4	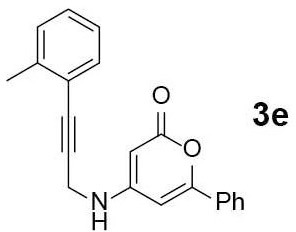	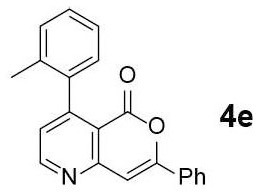	2	48
5	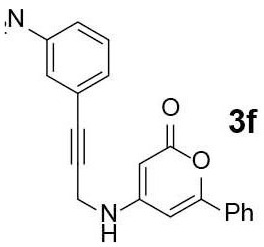	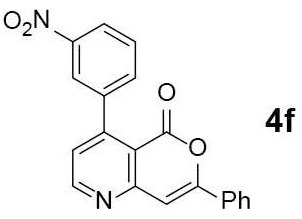	2	52^a^
6	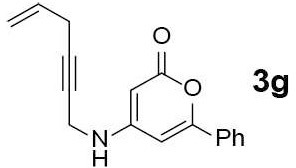	–	3	Decomposed
7	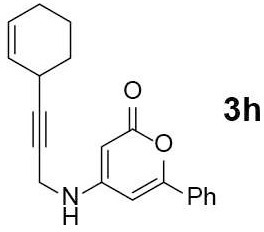	–	2	Decomposed
8	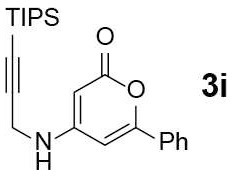	–	6	NR^b^

a Two steps yield from **2**;

b *Starting material was recovered*.

## Conclusion

In conclusion, an alternative synthetic route for 3-aryl-5-azaisocoumarins was developed which includes novel approach to install pyridine moieties on a readily available phenyl-2-pyrone precursor via regioselective metal-catalyzed cycloisomerization. Through an intensive preliminary investigation, AgOTf was determined to be an adequate alkyne-activation catalyst and was applicable in rearrangement of *N*-pyranonyl propargylamines bearing methyl or phenyl substituents to C-6 or C-8 substituted 3-aryl-5-azaisocoumarin. Derivatization of the pyridine moiety was accomplished efficiently via three-step reaction using the common starting material hydroxy-2-pyrone **1** and known propargylamines. Considering that 3-phenyl-5-azaisocoumarin **4a**, which was synthesized previously as a regioisomeric mixture using conventional lactonization strategies, was obtained regioselectively in moderate yield, this novel step-economical procedure can be widely utilized in derivatization of C-3, C-6, and C-8 of 5-azaisocoumarins. Further studies on the synthetic applications of the newly developed method are currently underway.

## Data Availability Statement

All datasets generated for this study are included in the article/Supplementary Material.

## Author Contributions

YH conceptualized the work. JY and CL synthesized all compound and performed m.p. R_f_, NMR, IR, LRMS, and HRMS analysis. The manuscript was written by CL and YH. All authors approved the manuscript in its final form for publication.

## Conflict of Interest

The authors declare that the research was conducted in the absence of any commercial or financial relationships that could be construed as a potential conflict of interest.
