# Supplementary material for: Preliminary Study on Novel Expedient Synthesis of 5-Azaisocoumarins by Transition Metal-Catalyzed Cycloisomerization
Source: Front Chem. 2020 Sep 4;8:772. doi: 10.3389/fchem.2020.00772 (PMC7500256; doi:10.3389/fchem.2020.00772)
Supplement: Supplementary file 1 [file Data_Sheet_1.DOCX]

Supplementary Material

**Novel Expedient Synthesis of 5-Azaisocoumarins by Transition Metal-Catalyzed Cycloisomerization**

Jeong A Yoon ^1^, Changjin Lim ^2, *^ and Young Taek Han ^1,*^

*^1^ College of Pharmacy, Dankook University, Cheonan 31116, Korea*

*^2^ School of Pharmacy, Jeonbuk National University, Jeonju 54896, Korea*

*** Correspondence:**Changjin Lim
limcj@jbnu.ac.kr

Young Taek Han
hanyt@dankook.ac.kr

**Table of Contents**

1. General Information ·························································································· S2

2. Preparation of Known Compounds ········································································· S3

3. ^1^H- and ^13^C-NMR Spectra ··················································································· S5

4. References ····································································································S19

# General Information

Unless noted otherwise, all starting materials and reagents were obtained commercially and were used without further purification. All solvents utilized for routine product isolation and chromatography were of reagent grade and glass-distilled, and reaction flasks were dried at 100 °C before use. Flash column chromatography was performed using silica gel 60 (230-400 mesh, Merck, Kenilworth, NJ, USA) with the indicated solvents. Thin-layer chromatography (TLC) was performed using 0.25-mm silica gel plates (Merck, Kenilworth, NJ, USA). Mass spectra were obtained using an Agilent 6530 Q-TOF (Santa Clara, CA, USA) instrument. Infrared spectra were recorded on a JASCO FT-IR-4200 spectrometer (Tokyo, Japan). ^1^H and ^13^C spectra were recorded on a Brucker Analytik ADVANCE digital 500 (500 MHz) (Billerica, MA, USA) and BRUKER AVANCE-800 (Billerica, MA, USA). Chemical shifts are expressed in parts per million (ppm, *δ*) downfield from tetramethylsilane and are referenced to the deuterated solvent. ^1^HNMR data are reported in the order of chemical shift, multiplicity (s, singlet; d, doublet; t, triplet; q, quartet; m, multiplet; bs, broad singlet; and/or multiple resonances), number of protons, and coupling constant in hertz (Hz).

# Preparation of Known Compounds

*4-Hydroxy-6-phenyl-2H-pyran-2-one* (**1**). **1** was prepared according to the literature procedure and the ^1^H-NMR spectra was consistent with the previous data (Prasad et al., 1995). Yellow solid of **1** (1.18 g, 82%) was obtained from 1 g of malonyl chloride. m.p.: 283–284 ˚C; R_f_ = 0.23, (MeOH/CH_2_Cl_2_ = 1 : 10); ^1^H-NMR (400 MHz, DMSO-d_6_) *δ* 11.89 (s, 1H, OH) 7.87–7.83 (m, 2H, Ar-H) 7.53–7.50 (m, 3H, Ar-H) 6.77 (d, 1H, *J* = 2.5 Hz, cyclic-CH) 5.40 (d, 1H, *J* = 2.5 Hz, cyclic-CH); ^13^C-NMR (125 MHz, DMSO-d_6_) *δ* 170.5, 163.0, 160.0, 131.0, 130.9, 129.1, 125.4, 98.4, 89.6 ; IR (thin film, neat) *ν*_max_ 1734, 1604, 1546, 1421, 1224, 1173, 1070, 804, 772 cm^-1^; LR-MS (ESI+) *m/z* 189 (M + H^+^); HR-MS (ESI+) calcd for C_11_H_9_O_3_ (M + H^+^) 189.0546; found 189.0544.

Structures of the synthesized propargylamine **3b’** (Nishizawa et al., 2010), **3c’** (Uredi et al., 2018), **3d’** (Sakthivel et al., 2017), **3e’** (Sakthivel et al., 2017), **3f’** (Uredi et al., 2019), **3g’** (Balducci et al., 2009), **3h’** (Cui et al., 2018), **3i’** (Valverde et al., 2009), **3j’** (Mons et al., 2019), and **3k’** (Sakthivel et al., 2017) were confirmed via comparison of their spectral data with those of the corresponding known propargylamine reported in the precedent literature.

**Table S1**. ^1^H NMR and ^13^C NMR assignments of 7-phenyl-5*H*-pyrano[4,3-*b*]pyridin-5-one **4a**

| Position | ^1^H-NMR | | ^13^C-NMR | |
| --- | --- | --- | --- | --- |
|  | Reported | Synthesized | Reported | Synthesized |
| 1 |  |  | 156.6 | 156.3 |
| 3 |  |  | 153.3 | 155.1 |
| 4 | 7.20 (s, 1H) | 7.23 (s, 1H) | 104.0 | 103.6 |
| 4a |  |  | 162.2 | 162.1 |
| 5 | 8.91 (dd, 1H, *J* = 1.3, 4.7 Hz) | 8.94 (dd, 1H, *J* = 1.8, 4.6 Hz) | 157.4 | 157.6 |
| 6 | 7.40  (dd, 1H, *J* = 4.7, 8.1 Hz), | 7.43  (dd, 1H, *J* = 4.6, 7.9 Hz) | 123.2 | 123.0 |
| 7 | 8.52 (dd, 1H, *J* = 1.3, 8.1 Hz) | 8.55  (dd, 1H, *J* = 1.4, 7.8 Hz) | 137.8 | 137.9 |
| 7a |  |  | 117.2 | 117.2 |
| 3’ |  |  | 131.6 | 131.5 |
| 4’, 8’ | 7.50-7.45 (m, 3H) | 7.51-7.47 (m, 3H) | 129.3 | 129.2 |
| 6’ |  |  |  |  |
|  |  |  | 125.9 | 125.9 |
| 5’, 7’ | 7.93-7.86 (m, 2H) | 7.93-7.91 (m, 2H) | 131.1 | 131.0 |

# ^1^H- and ^13^C-NMR Spectra

| ^1^H-NMR Spectra of **1** (400 MHz, DMSO-d_6_)  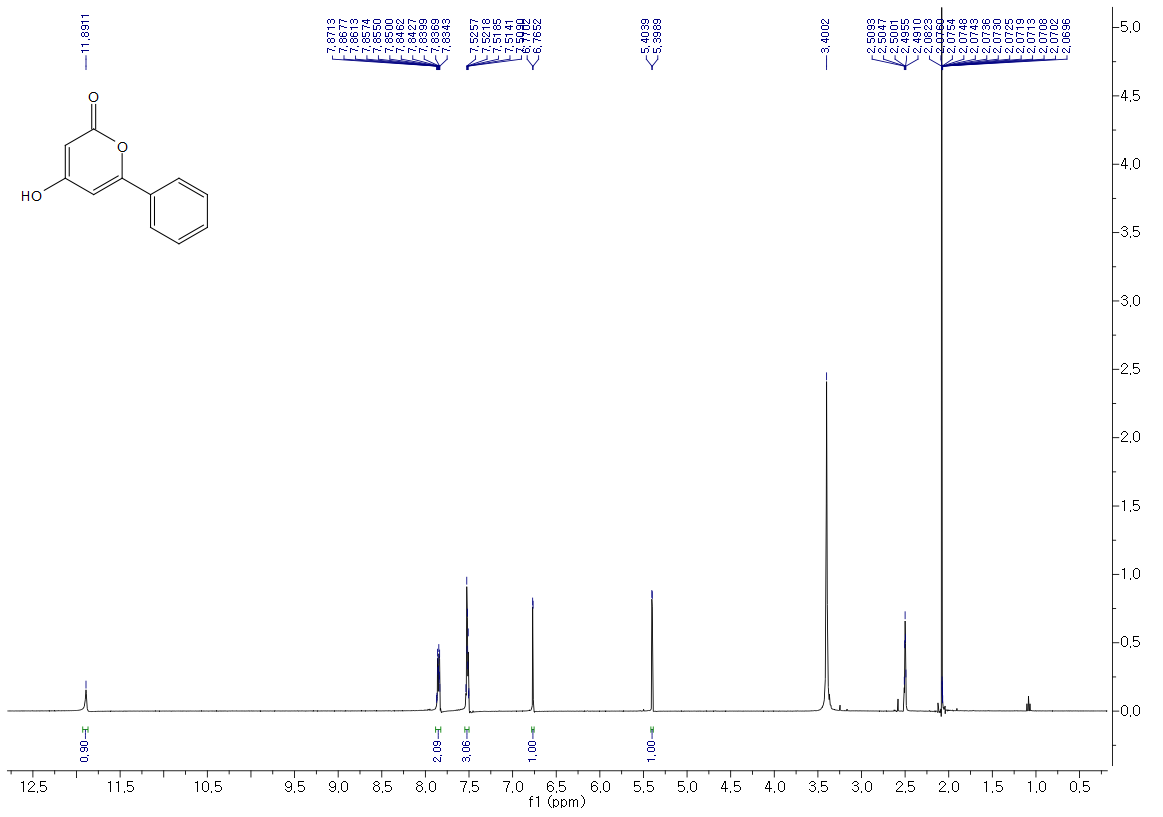 |
| --- |
| ^13^C-NMR Spectra of **1** (125 MHz, DMSO-d_6_)  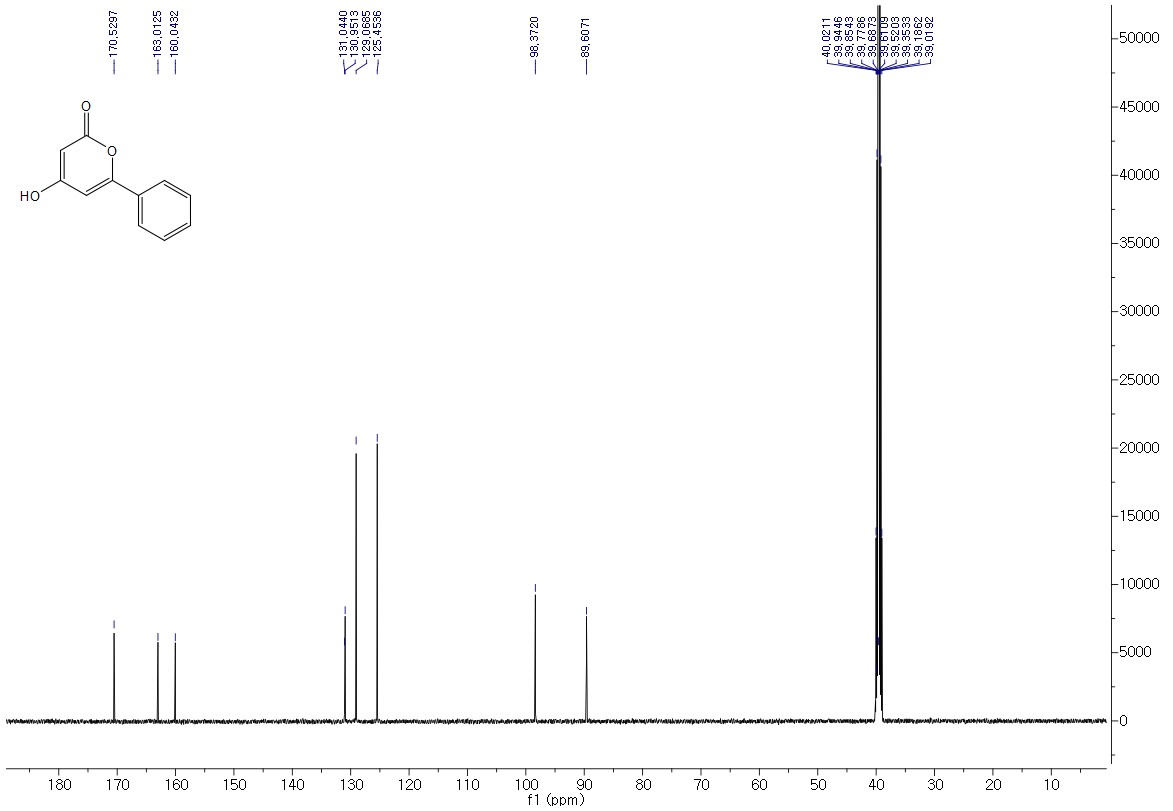 |
| ^1^H-NMR Spectra of **2** (500 MHz, CDCl_3_)  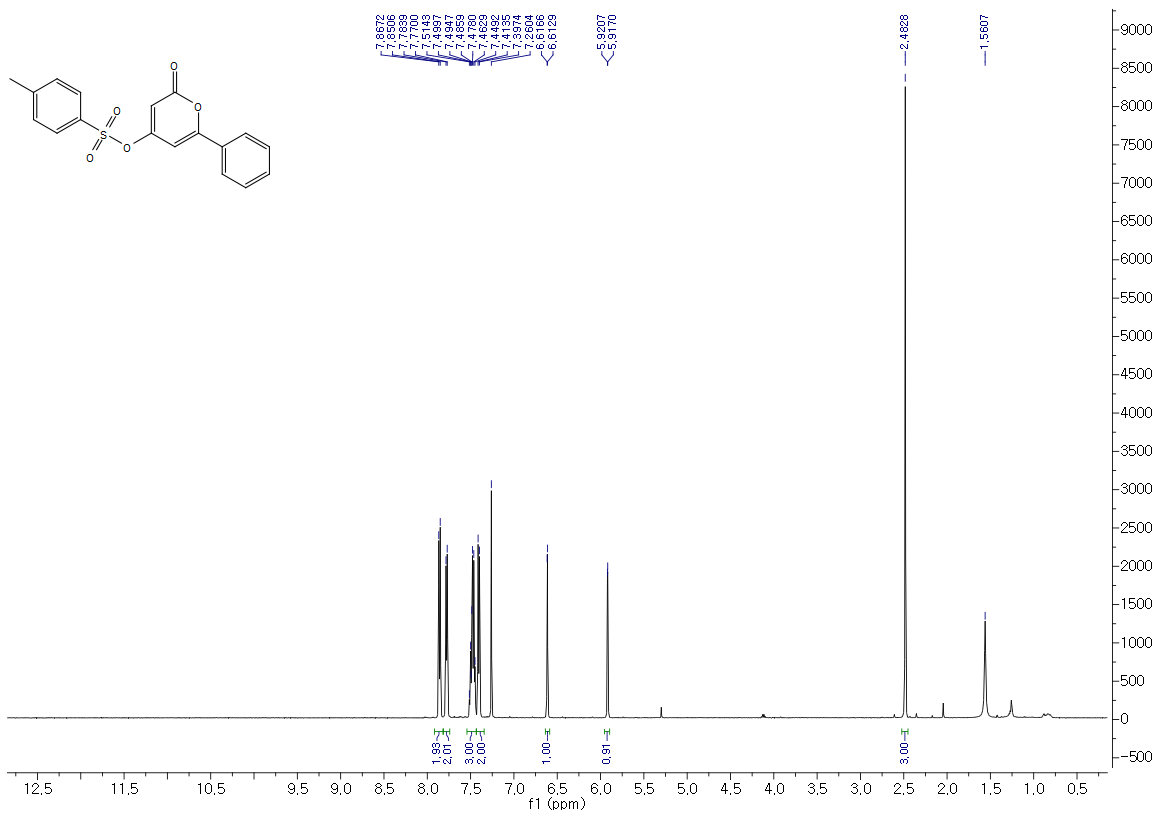 |
| ^13^C-NMR Spectra of **2** (125 MHz, CDCl_3_)  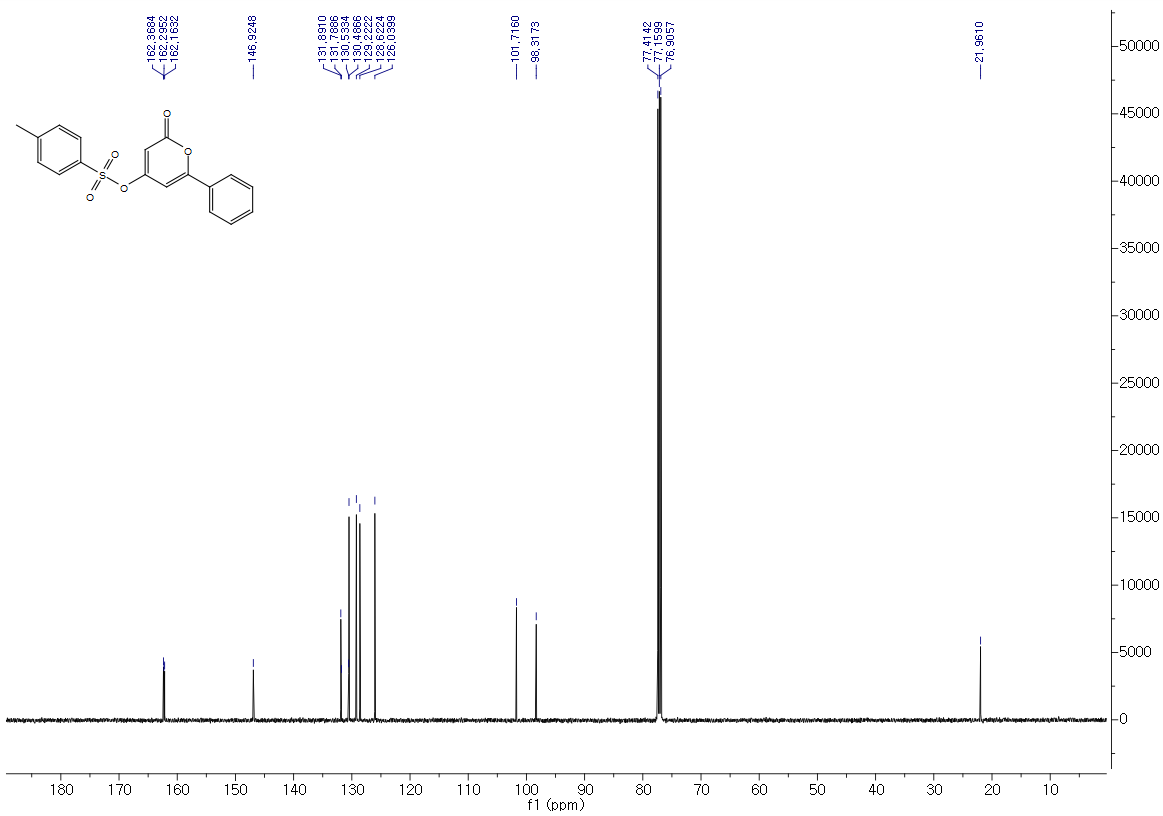 |
| ^1^H-NMR Spectra of **3a** (500 MHz, DMSO-d_6_)  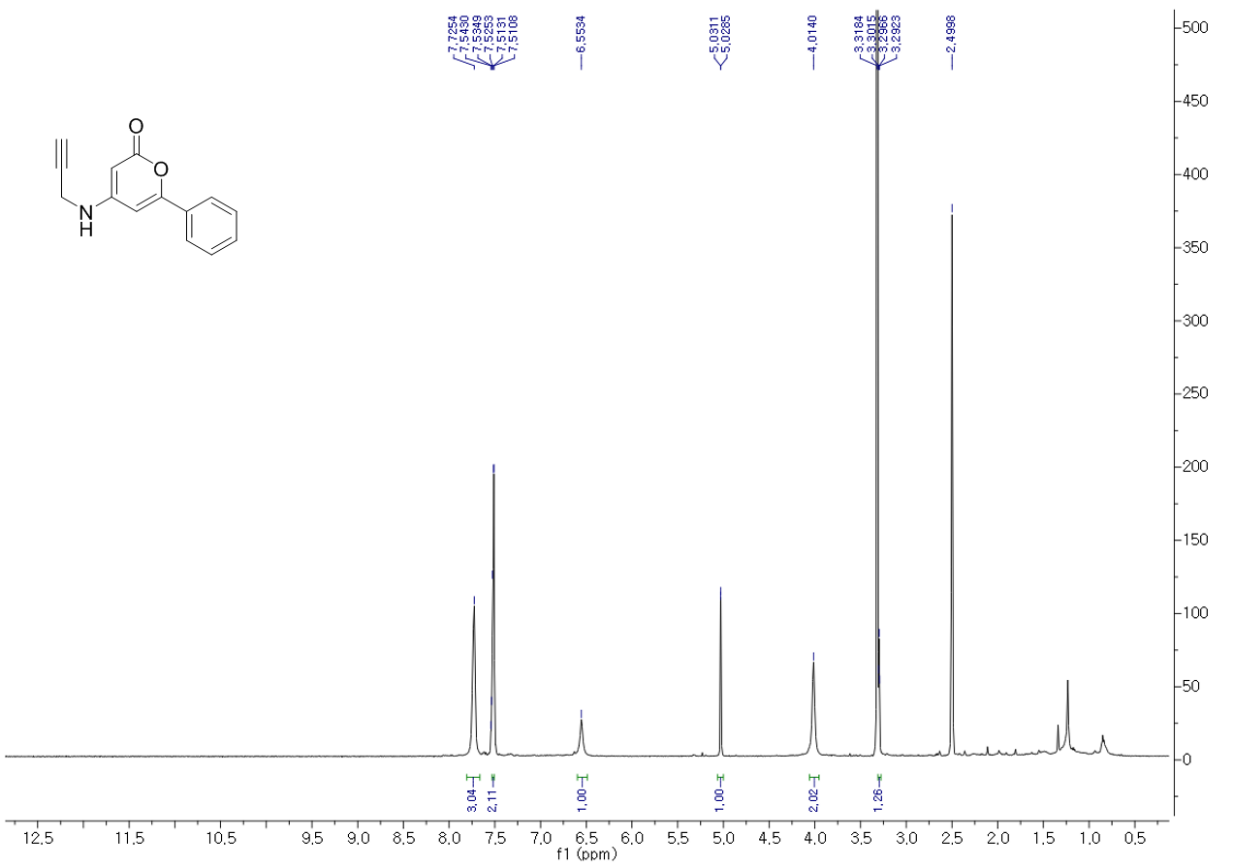 |
| ^13^C-NMR Spectra of **3a** (125 MHz, DMSO-d_6_)  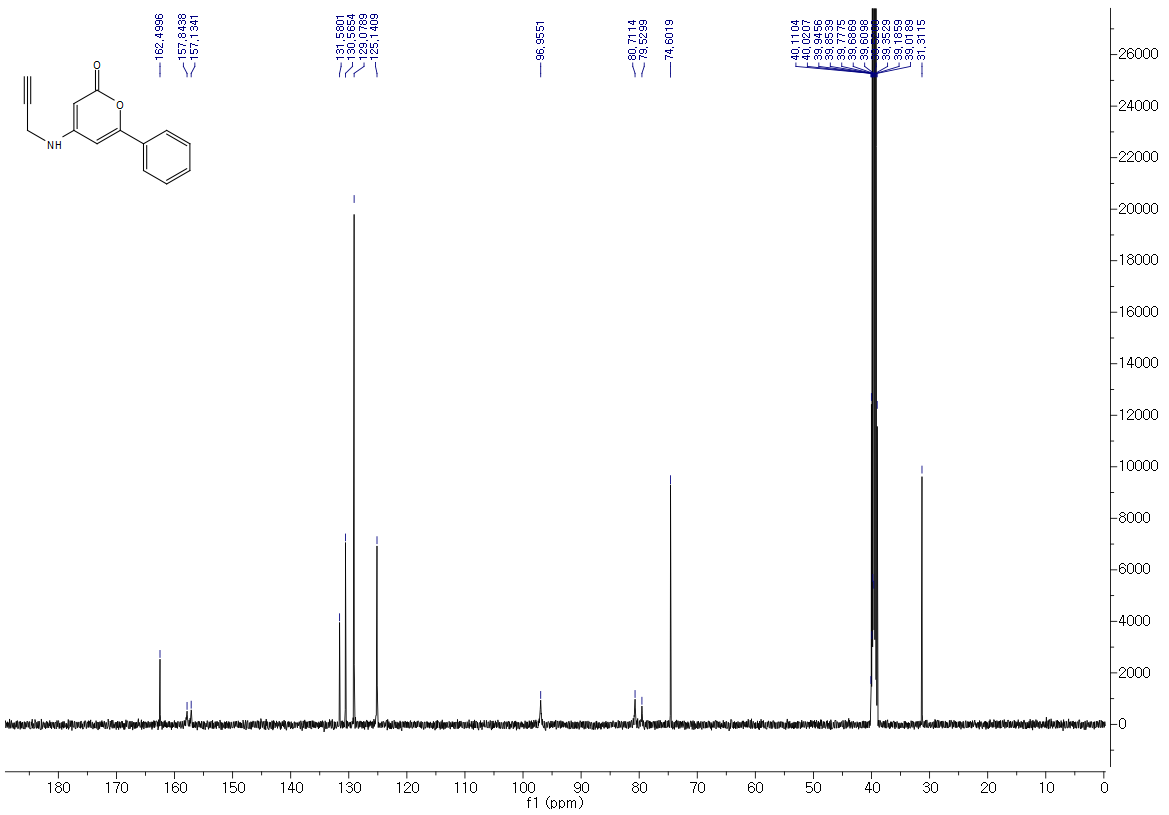 |
| ^1^H-NMR Spectra of **3b** (500 MHz, DMSO-d_6_)  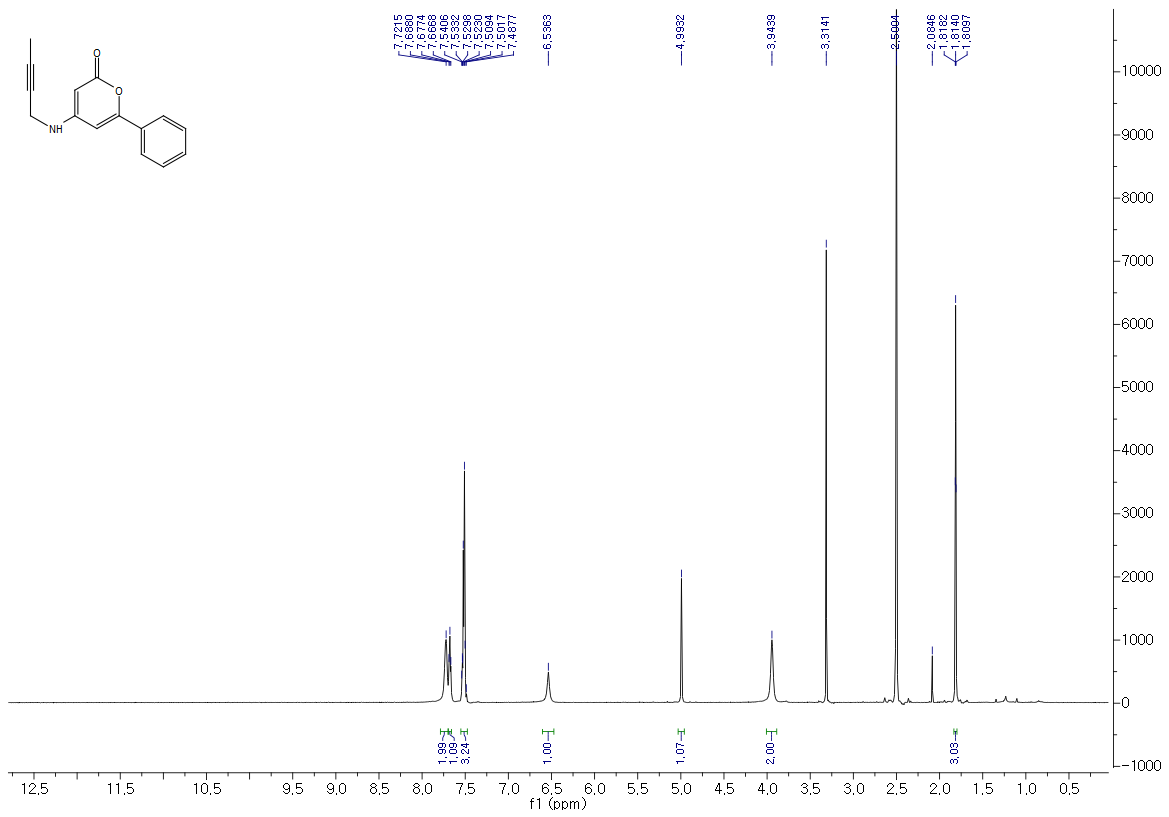 |
| ^13^C-NMR Spectra of **3b** (125 MHz, DMSO-d_6_)  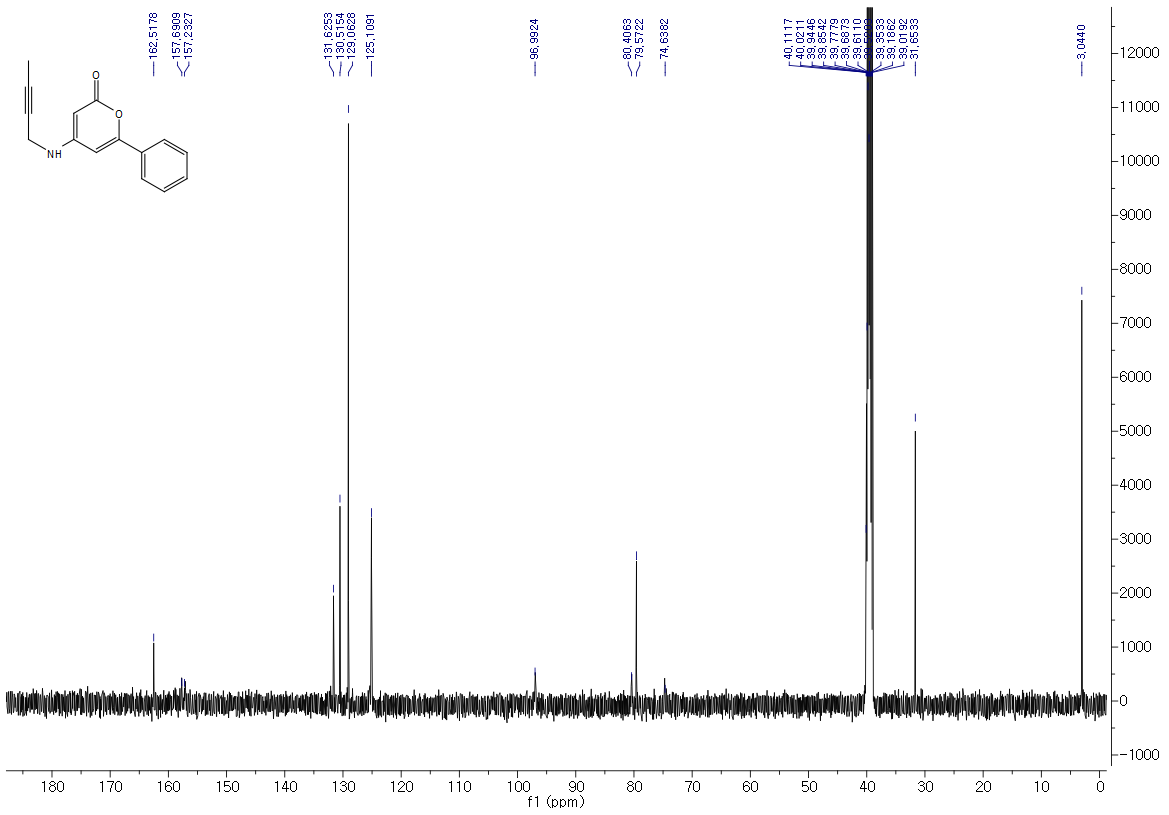 |
| ^1^H-NMR Spectra of **3c** (500 MHz, DMSO-d_6_)  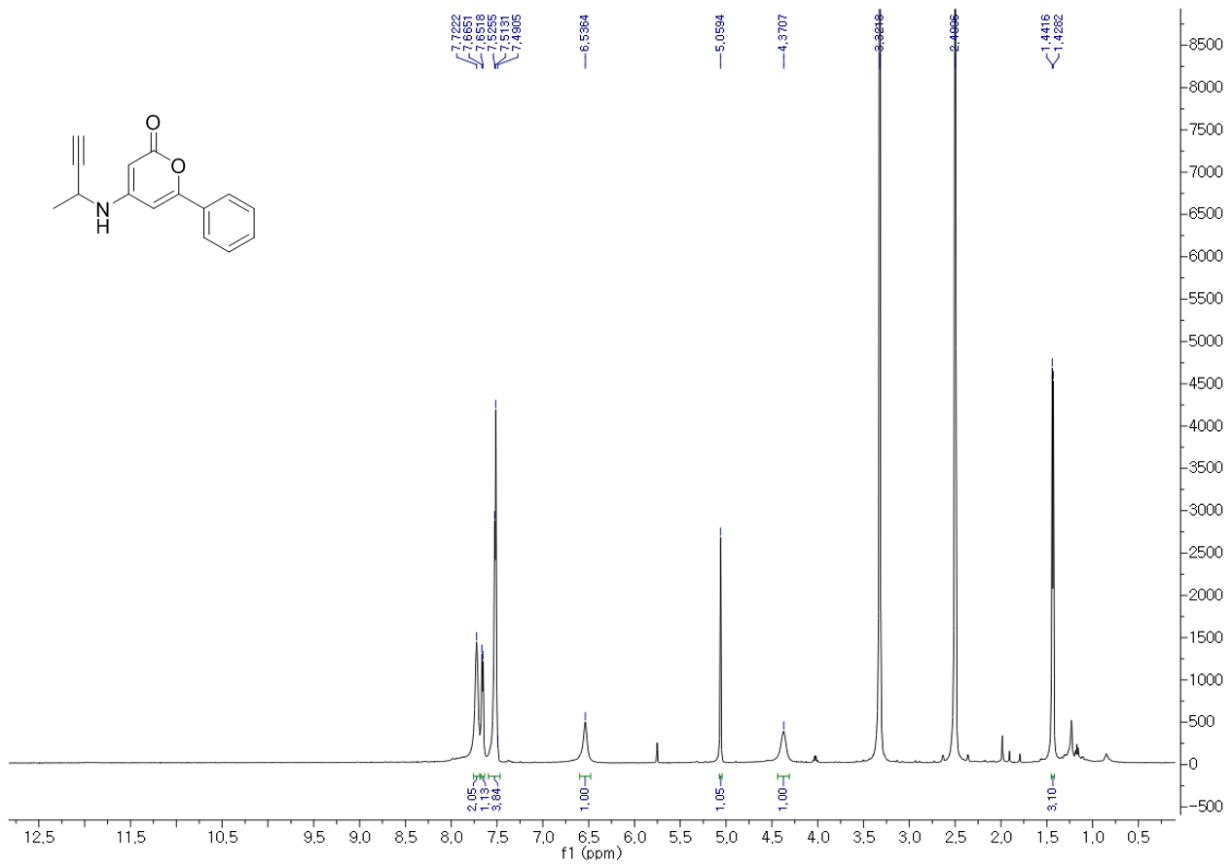 |
| ^13^C-NMR Spectra of **3c** (125 MHz, DMSO-d_6_)  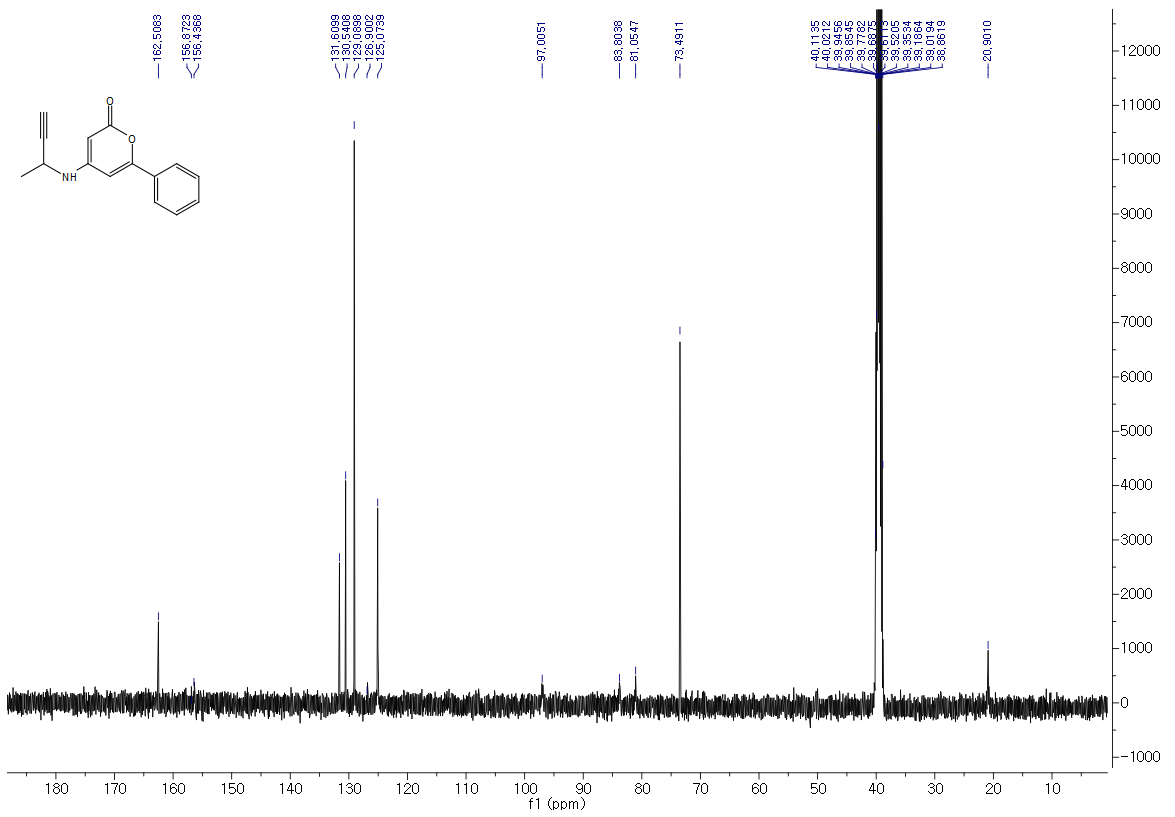 |
| ^1^H-NMR Spectra of **3d** (500 MHz, DMSO-d_6_)  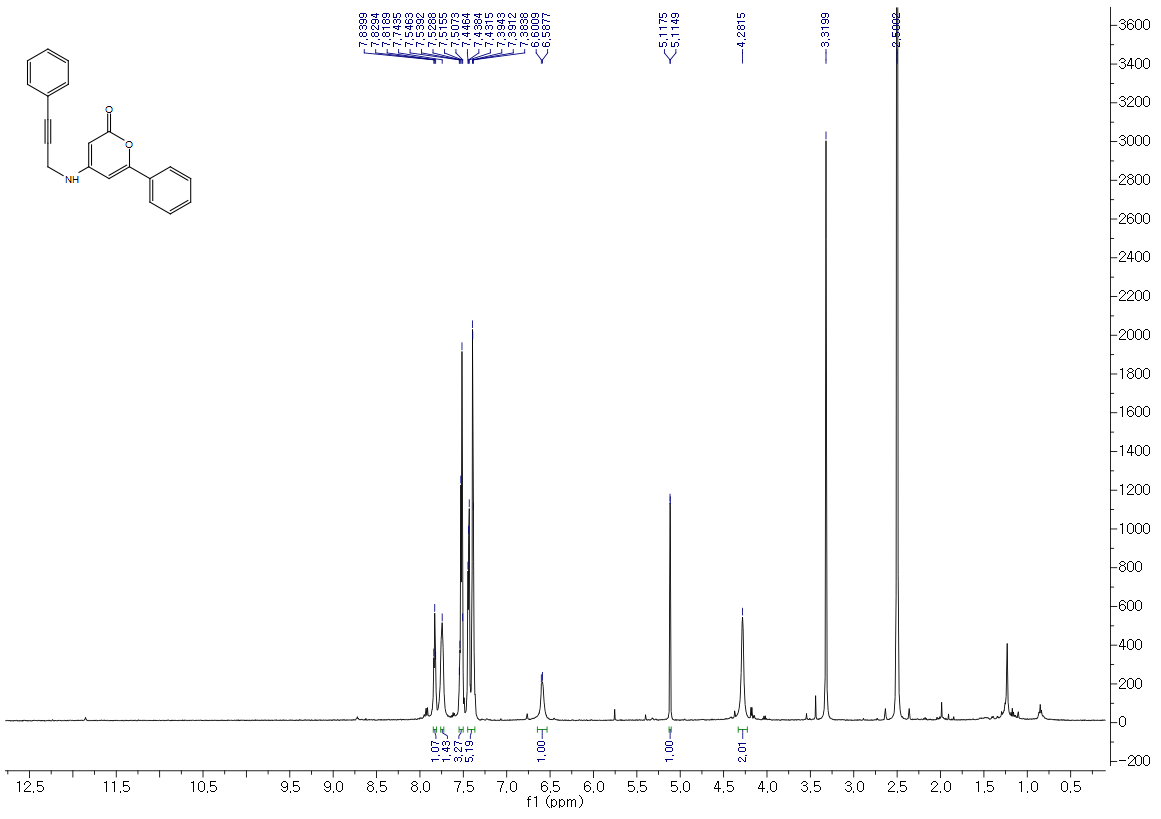 |
| ^13^C-NMR Spectra of **3d** (125 MHz, DMSO-d_6_)  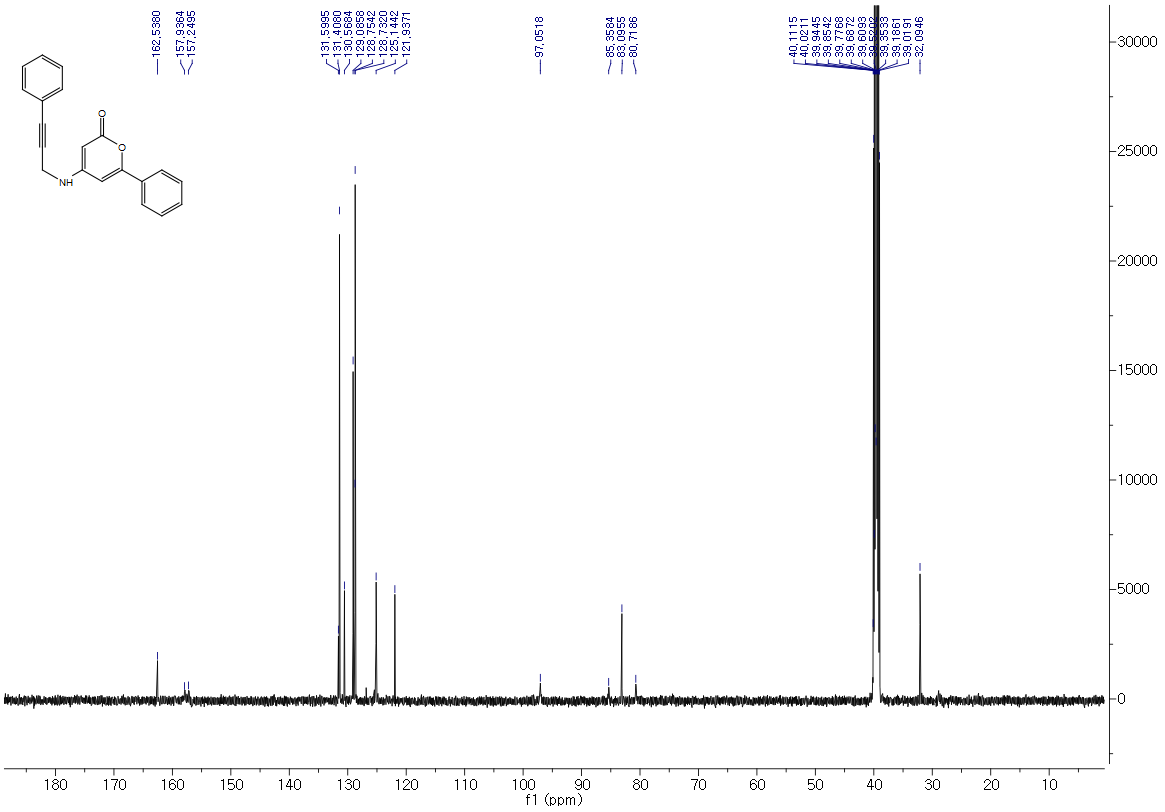 |
| ^1^H-NMR Spectra of **3e** (800 MHz, DMSO-d_6_)  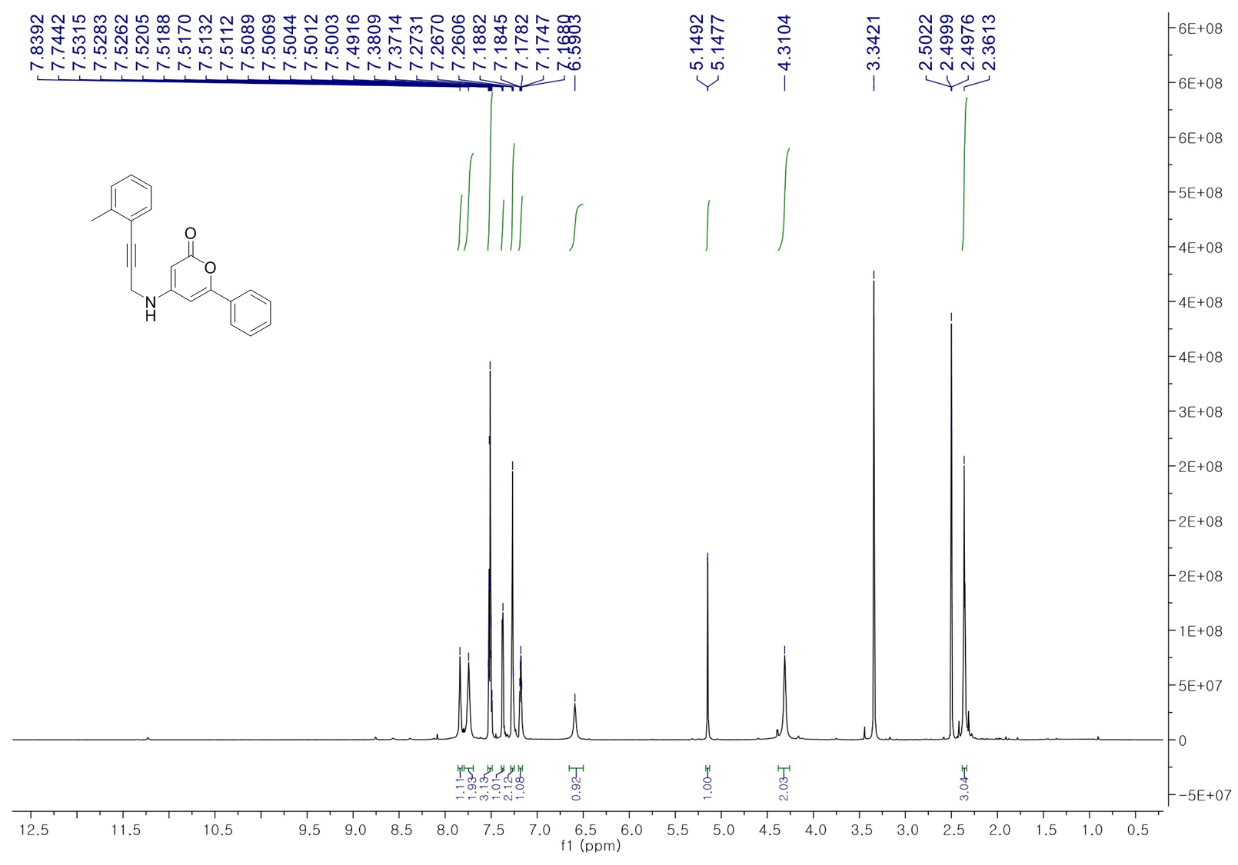 |
| ^13^C-NMR Spectra of **3e** (200 MHz, DMSO-d_6_)  **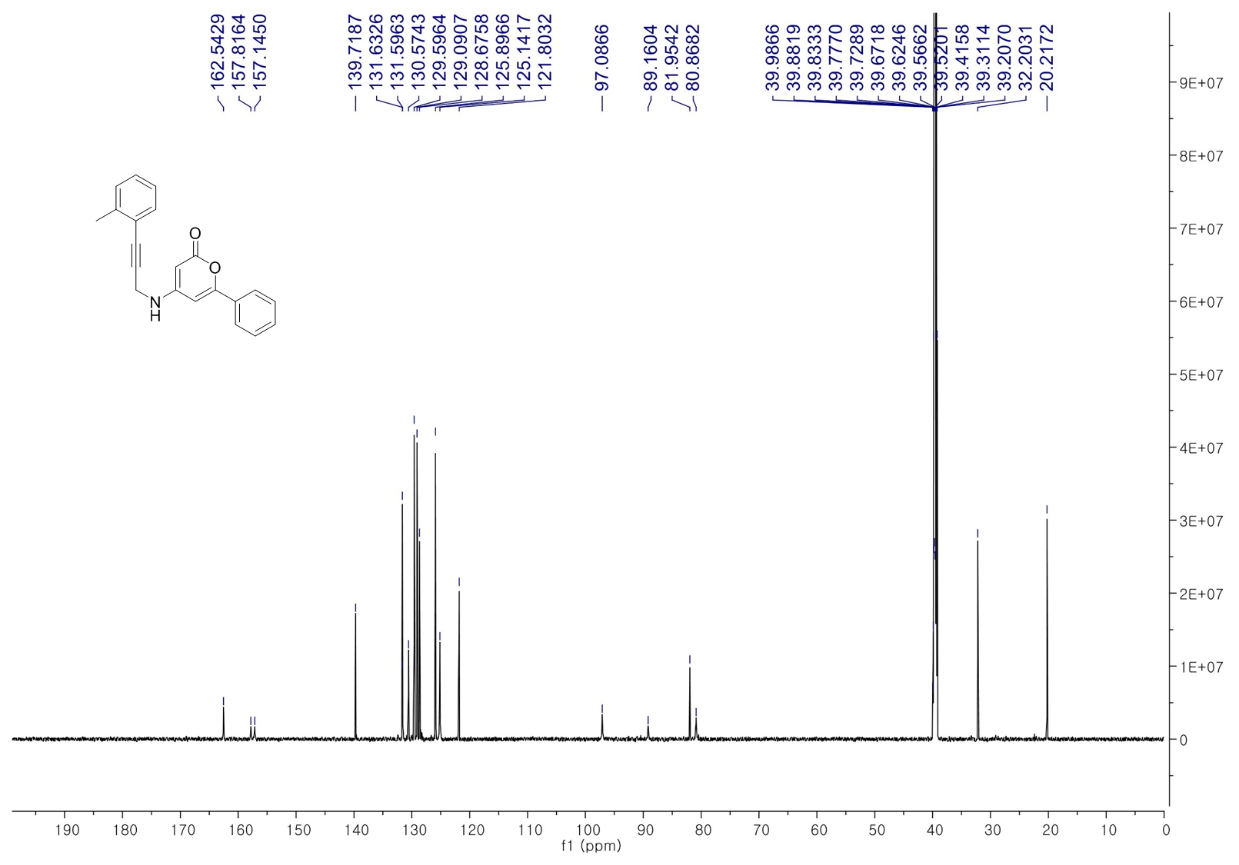** |
| ^1^H-NMR Spectra of **3g** (800 MHz, DMSO-d_6_)  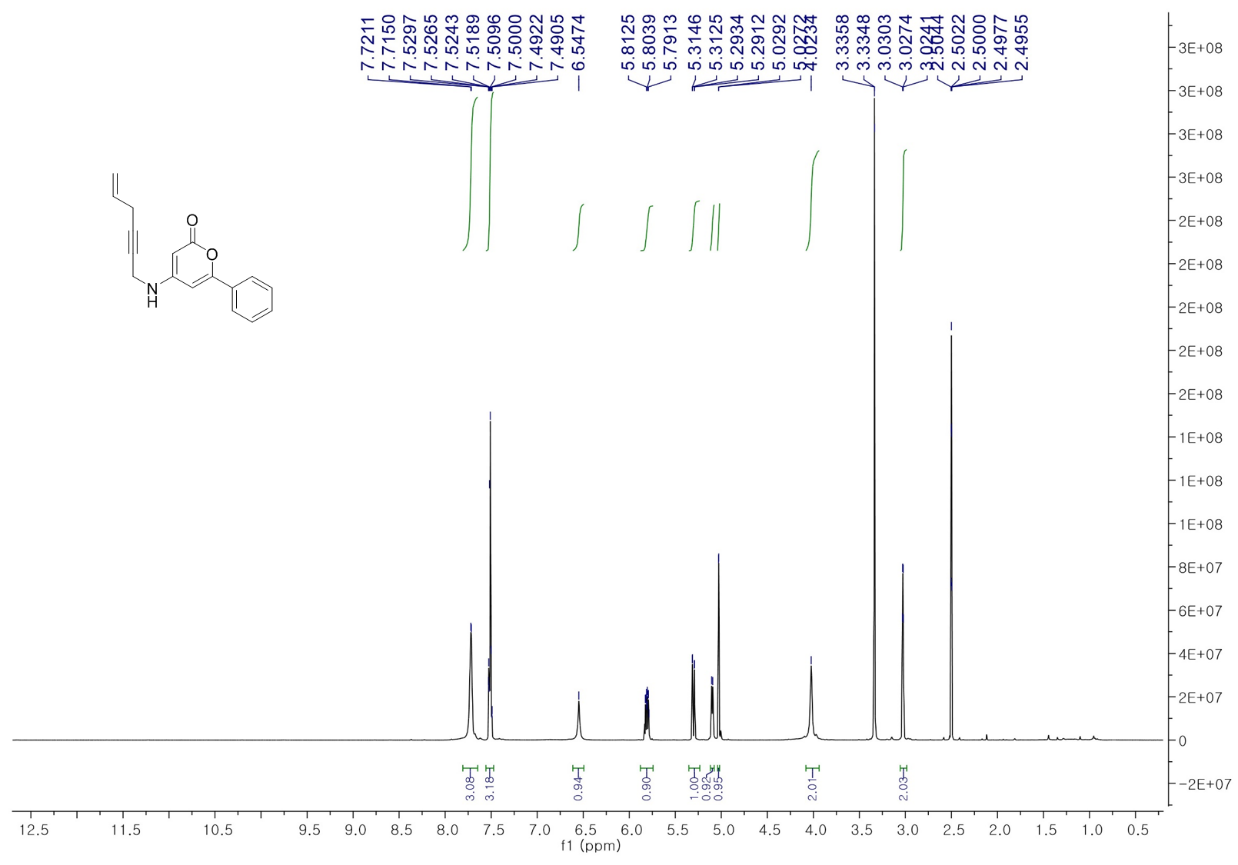 |
| ^13^C-NMR Spectra of **3g** (200 MHz, DMSO-d_6_)  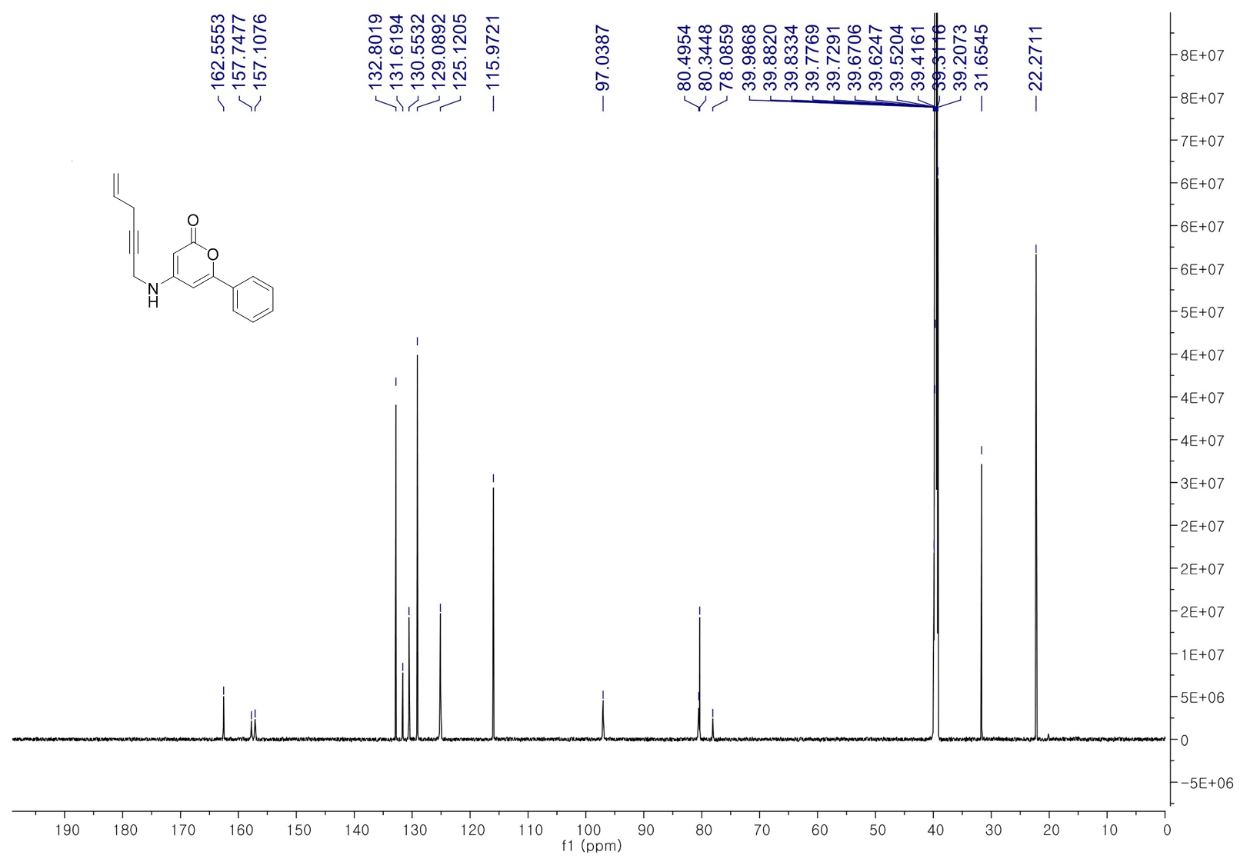 |
| ^1^H-NMR Spectra of **3h** (800 MHz, DMSO-d_6_)  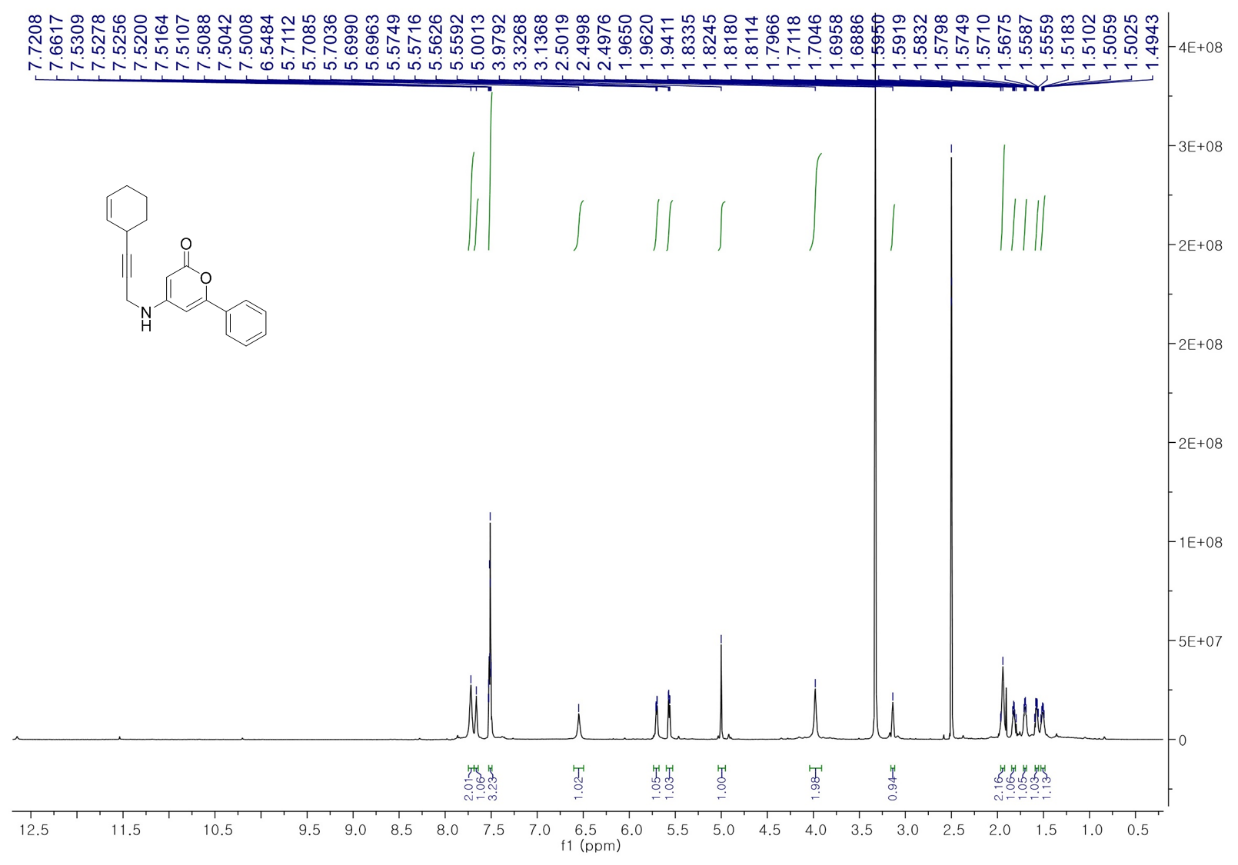 |
| ^13^C-NMR Spectra of **3h** (200 MHz, DMSO-d_6_)  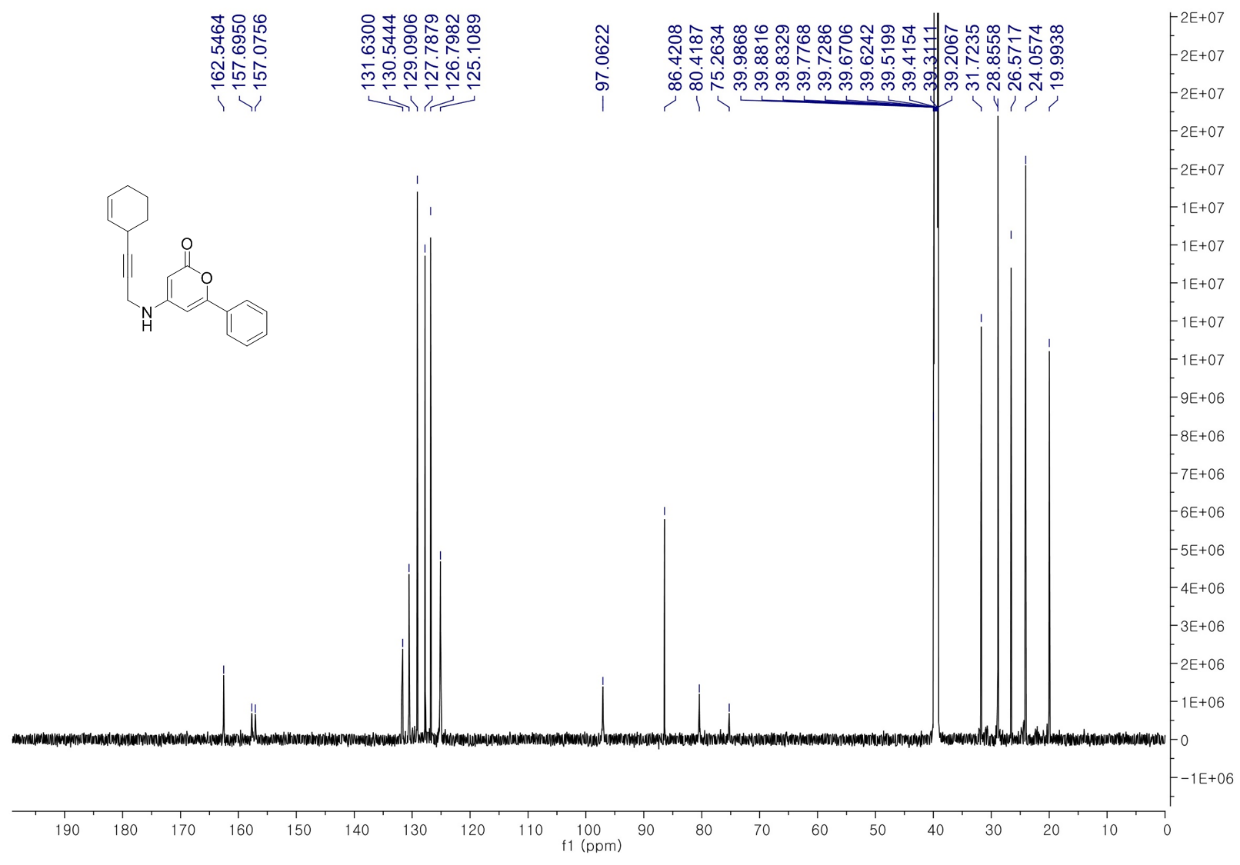 |
| ^1^H-NMR Spectra of **3i** (800 MHz, DMSO-d_6_)  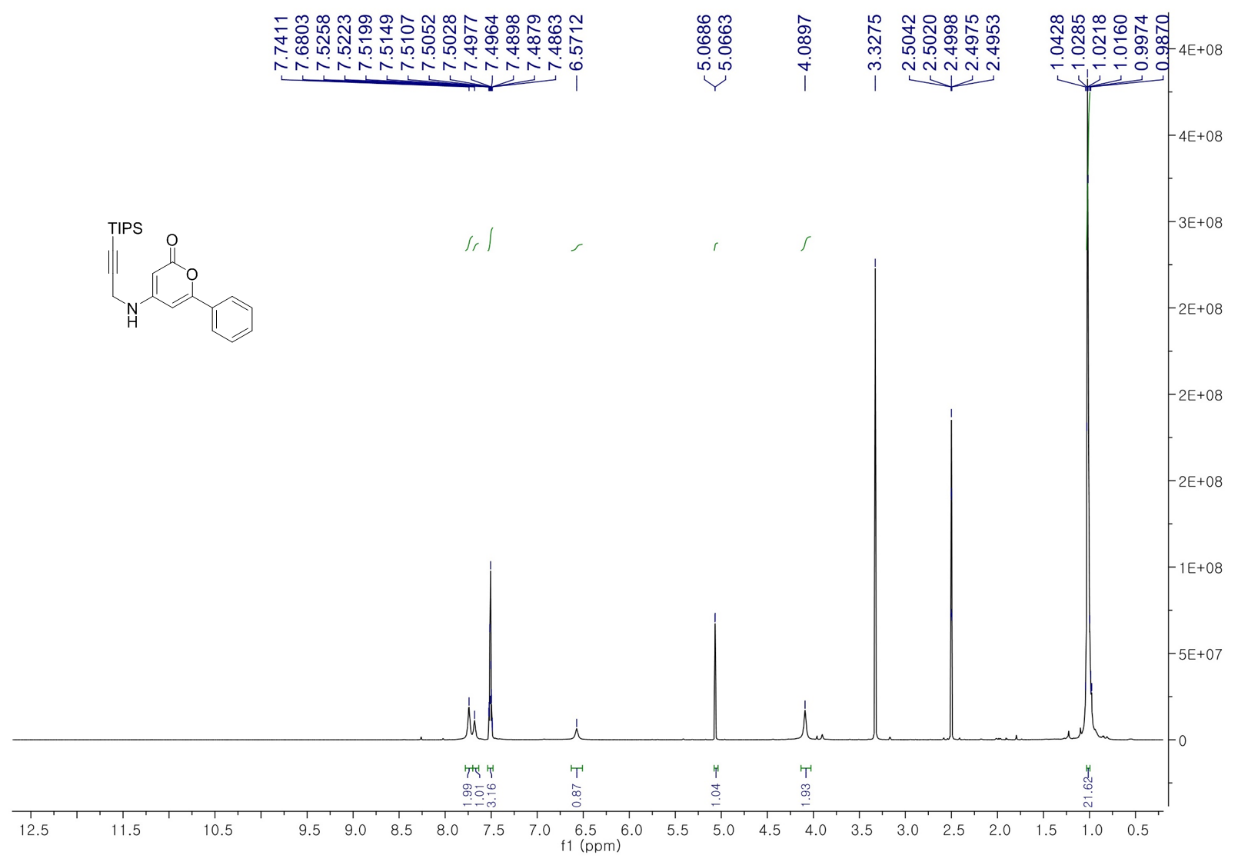 |
| ^13^C-NMR Spectra of **3i** (200 MHz, DMSO-d_6_)  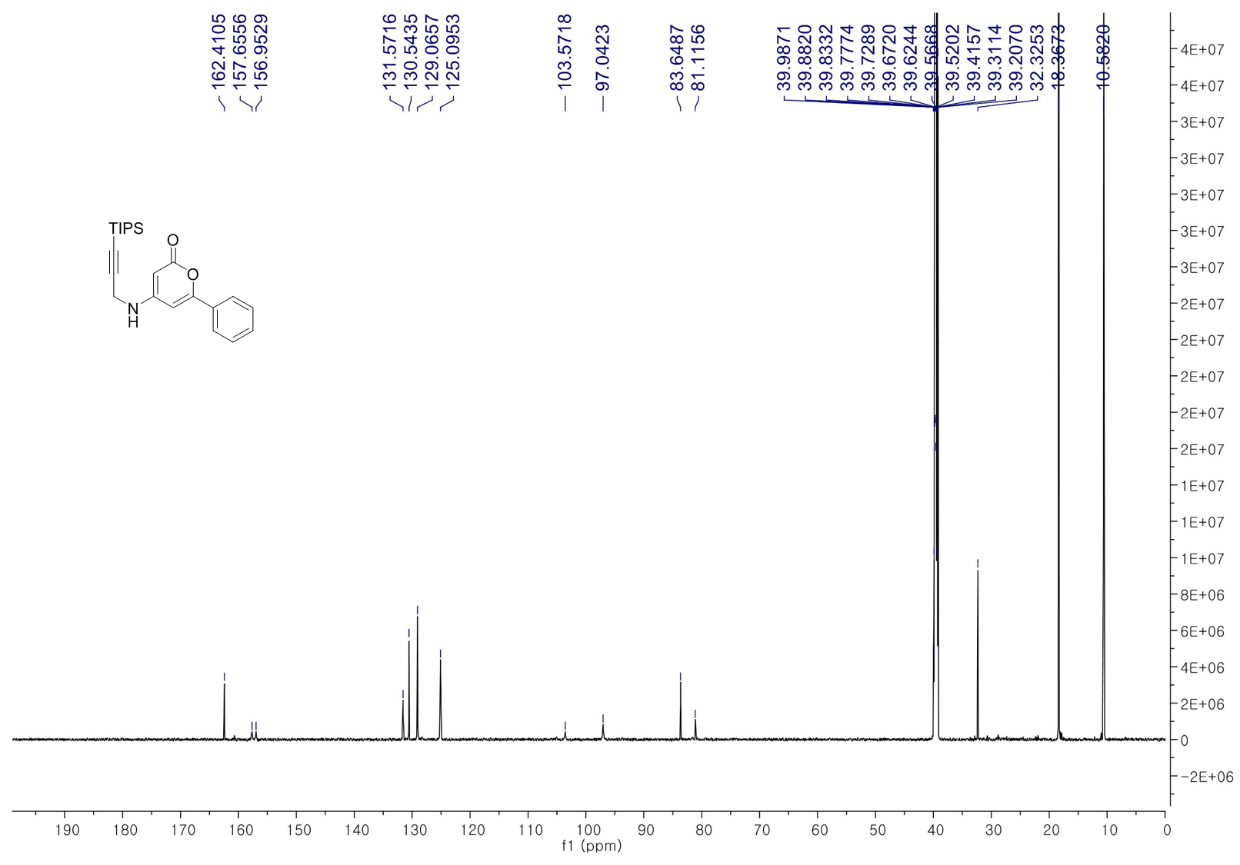 |
| ^1^H-NMR Spectra of **4a** (800 MHz, CDCl_3_)  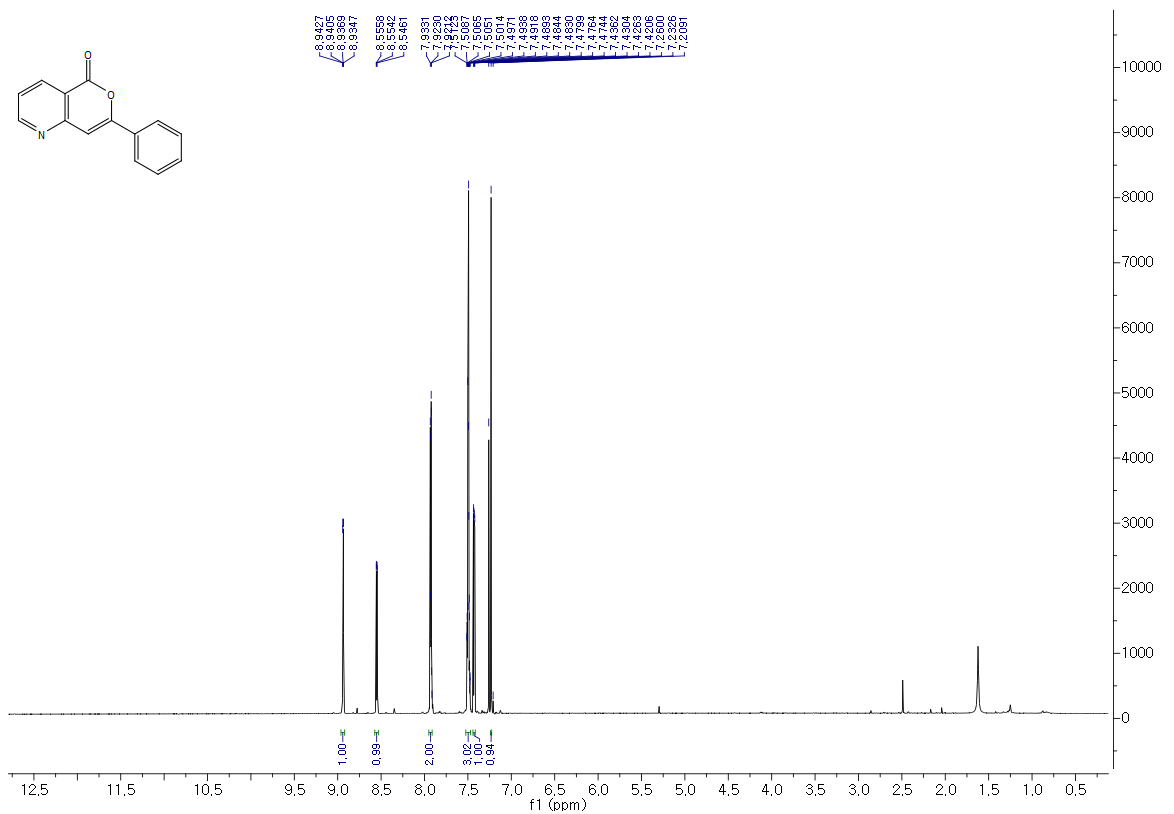 |
| ^13^C-NMR Spectra of **4a** (125 MHz, CDCl_3_)  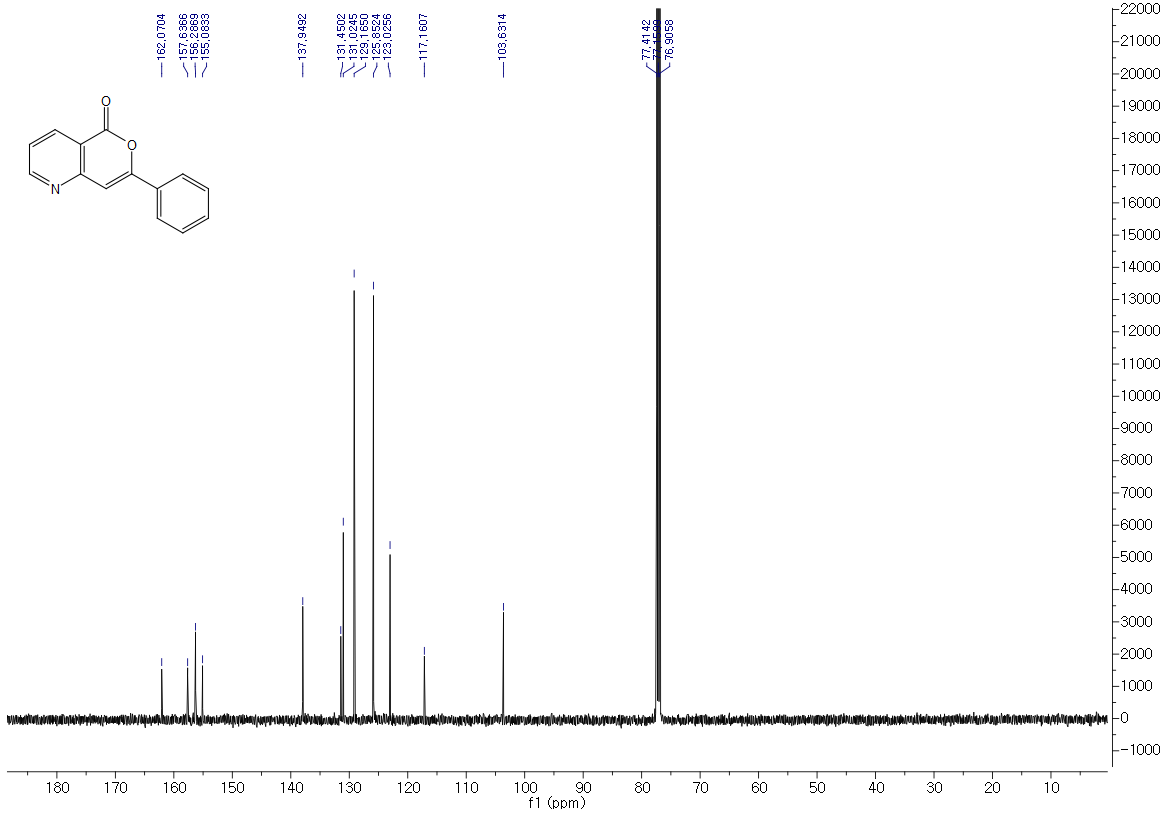 |
| ^1^H-NMR Spectra of **4b** (500 MHz, CDCl_3_)  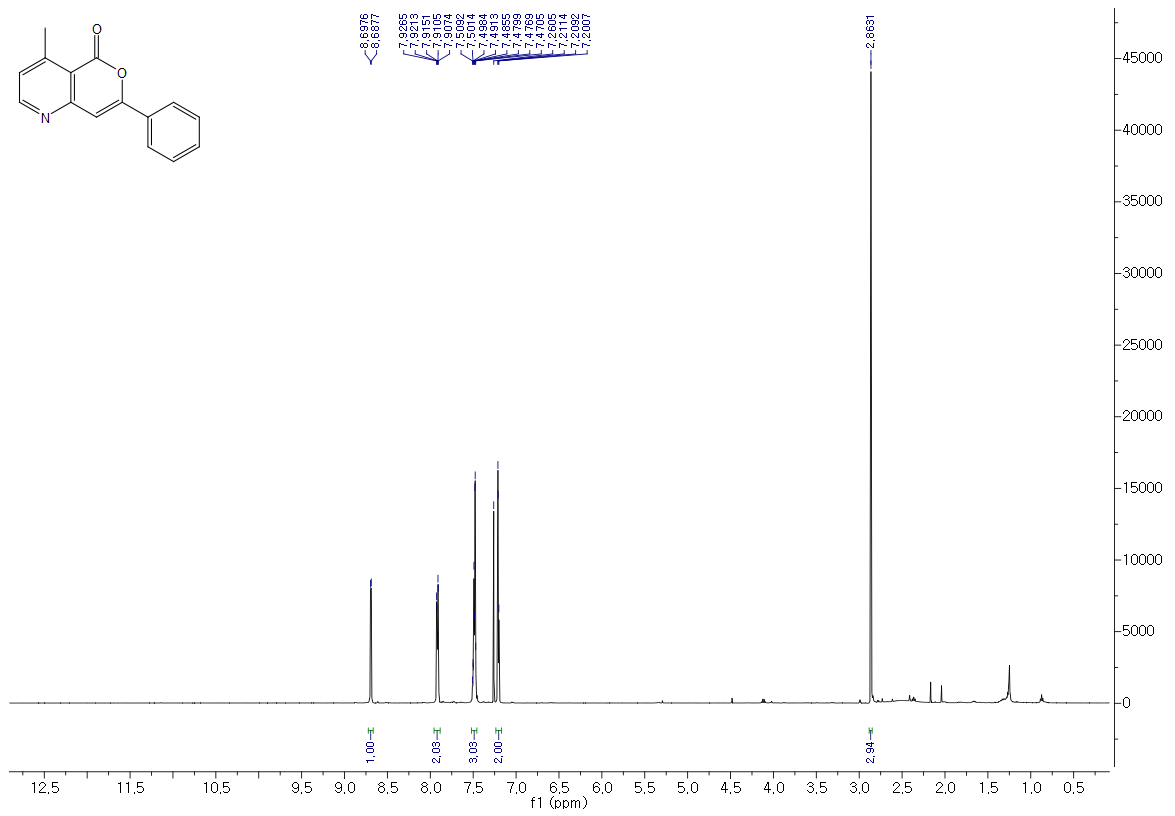 |
| ^13^C-NMR Spectra of **4b** (125 MHz, CDCl_3_)  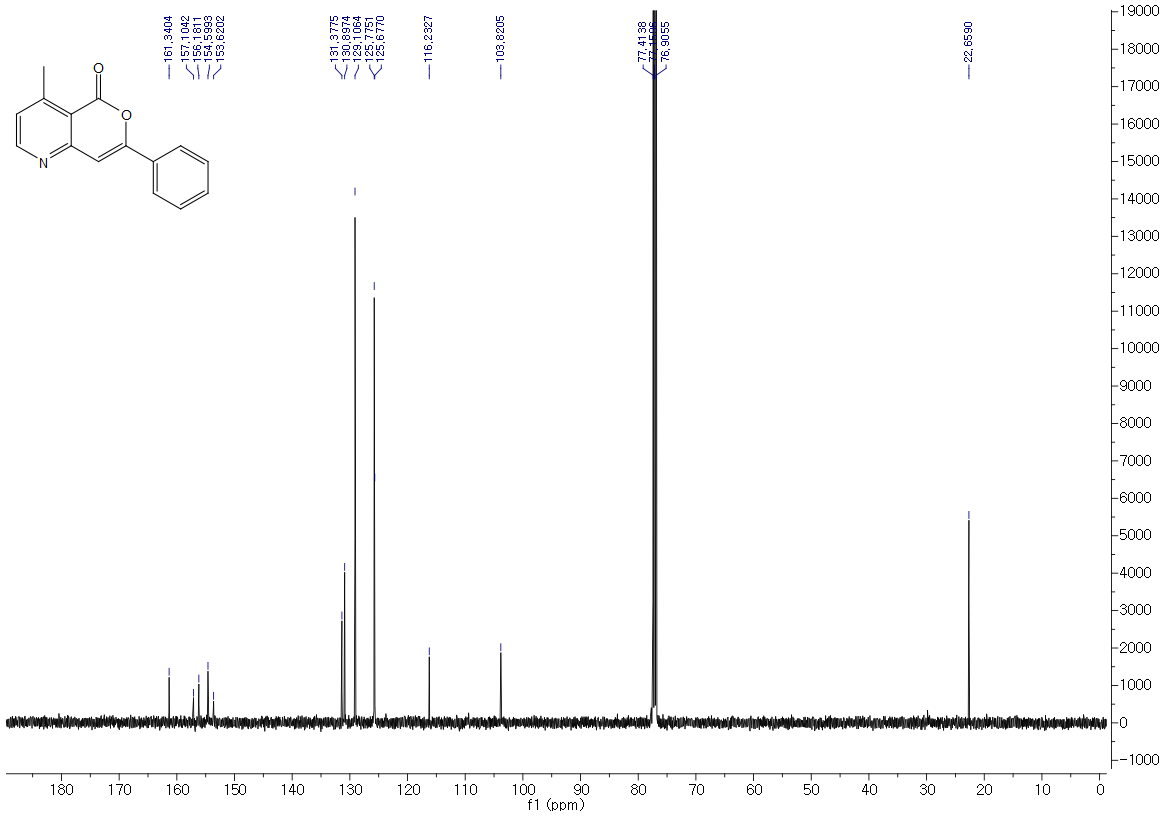 |
| ^1^H-NMR Spectra of **4c** (500 MHz, CDCl_3_)  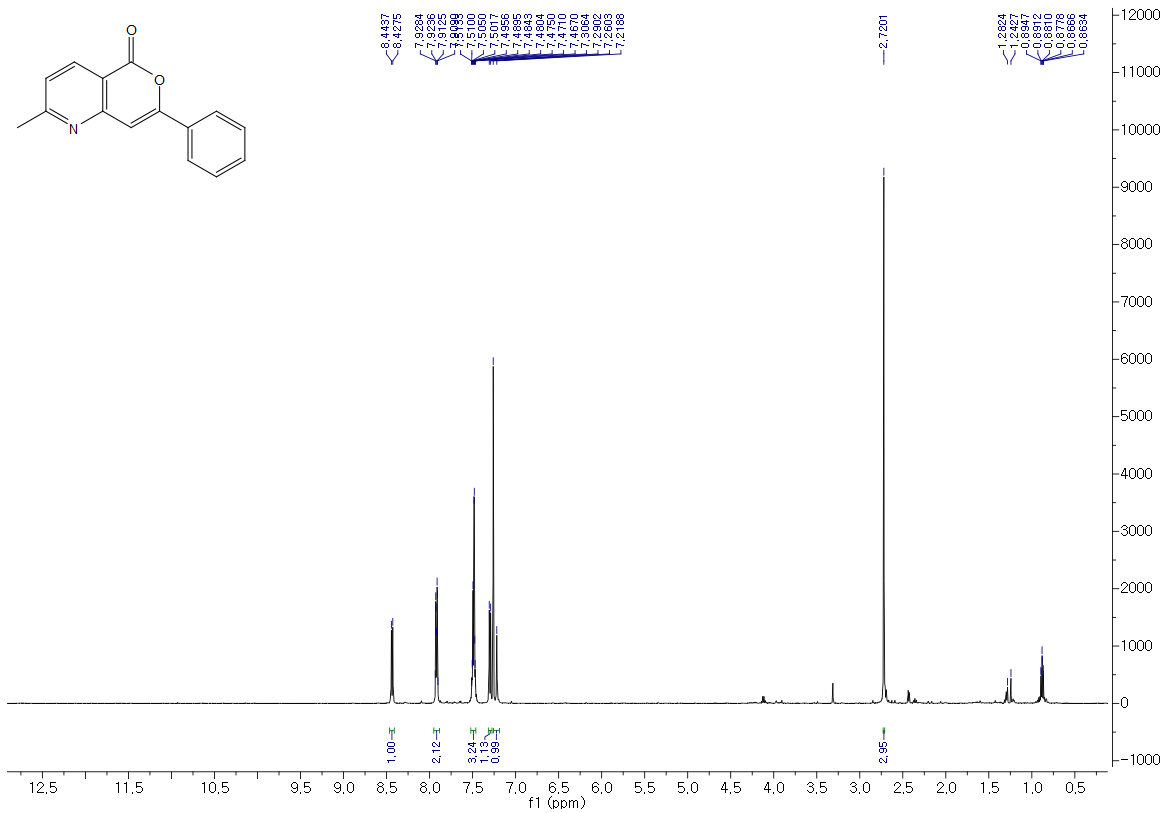 |
| ^13^C-NMR Spectra of **4c** (125 MHz, CDCl_3_)  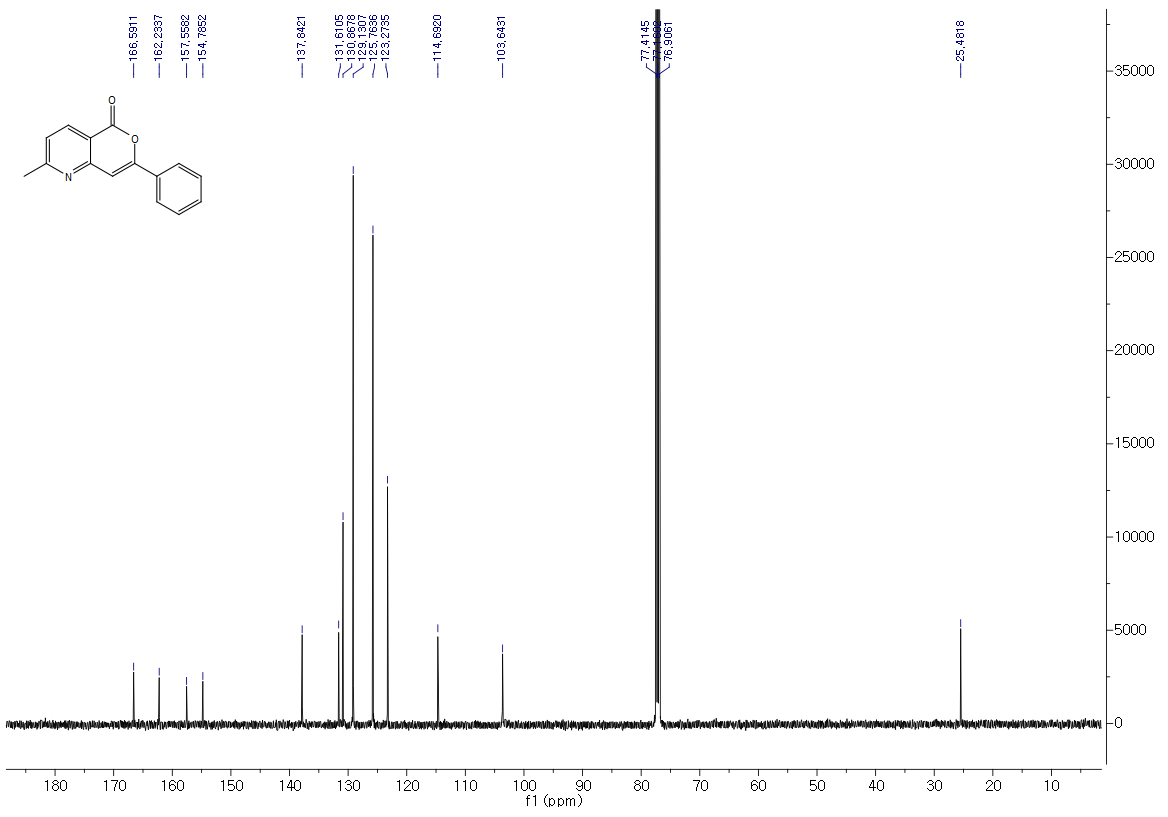 |
| ^1^H-NMR Spectra of **4d** (500 MHz, CDCl_3_)  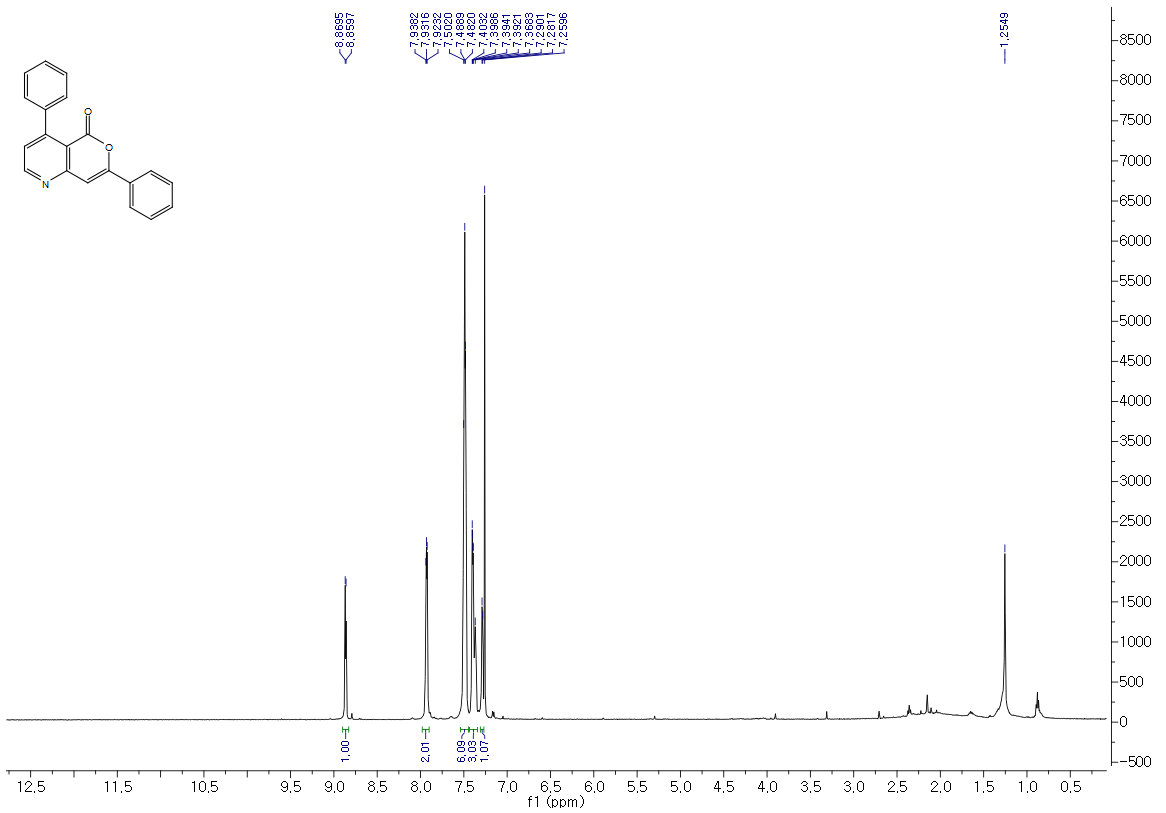 |
| ^13^C-NMR Spectra of **4d** (125 MHz, CDCl_3_)  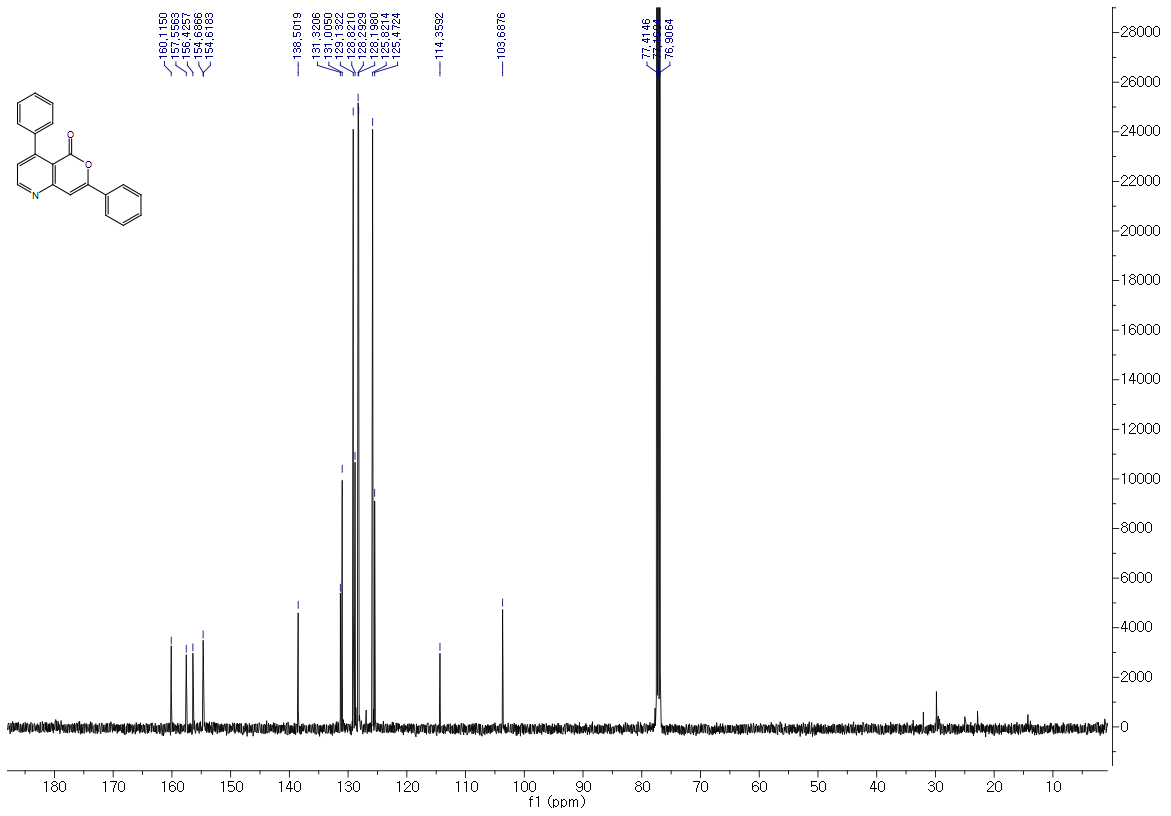 |
| ^1^H-NMR Spectra of **4e** (800 MHz, CDCl_3_)  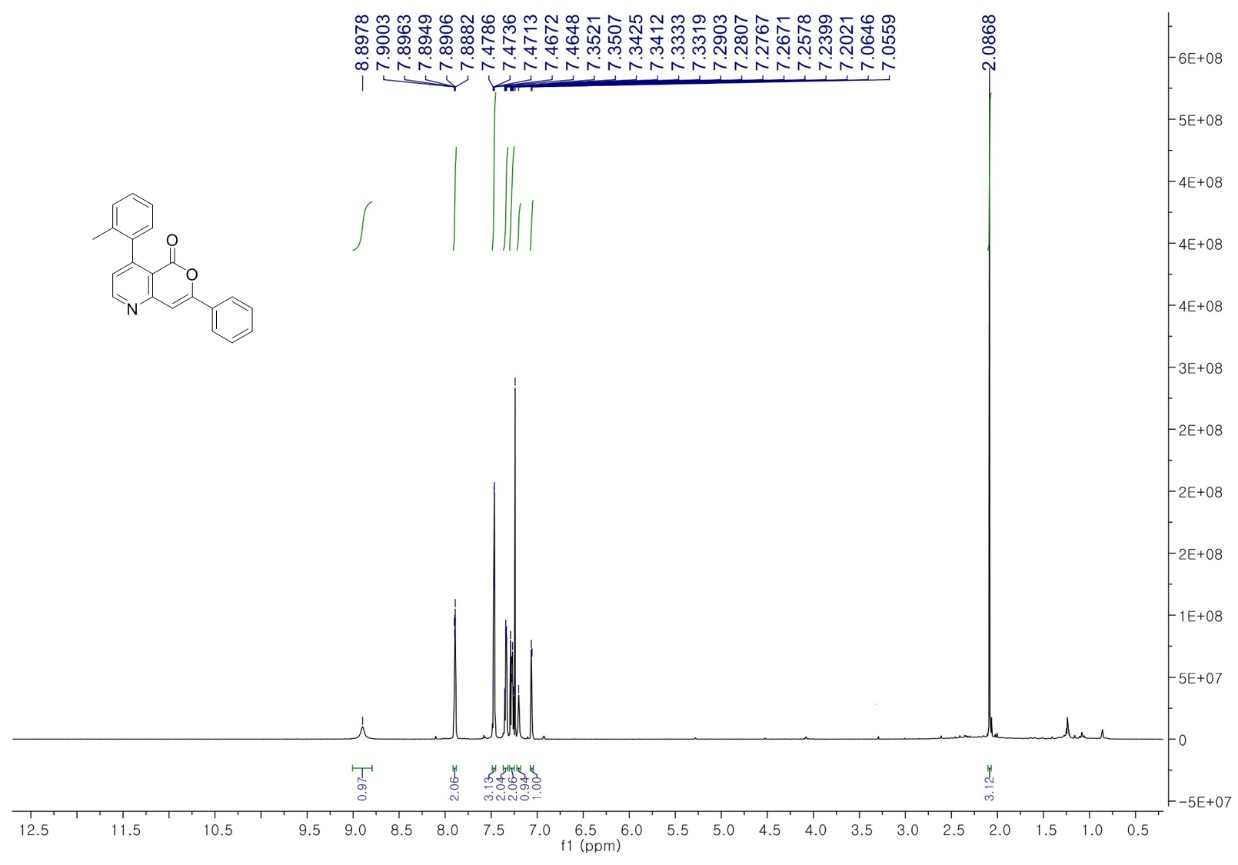 |
| ^13^C-NMR Spectra of **4e** (200 MHz, CDCl_3_)  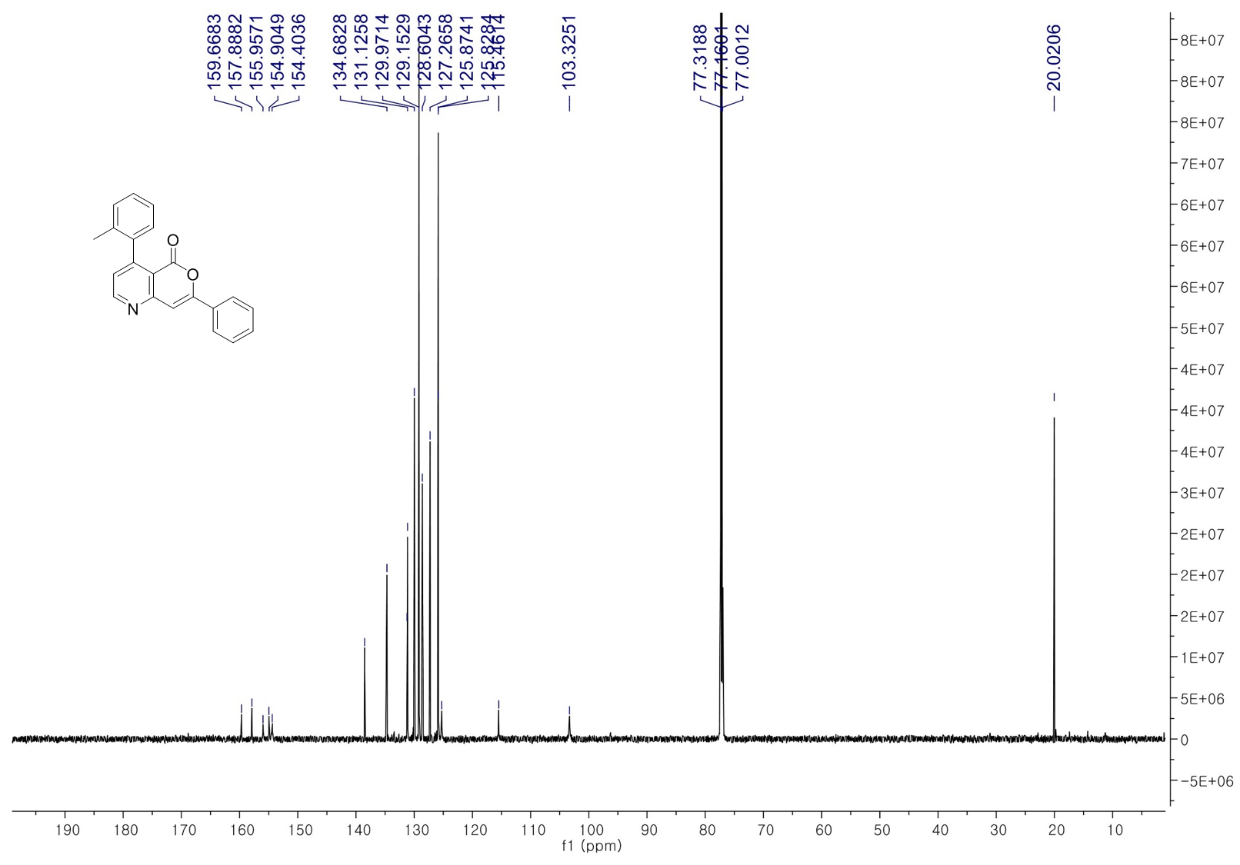 |
| ^1^H-NMR Spectra of **4f** (800 MHz, DMSO-d_6_)  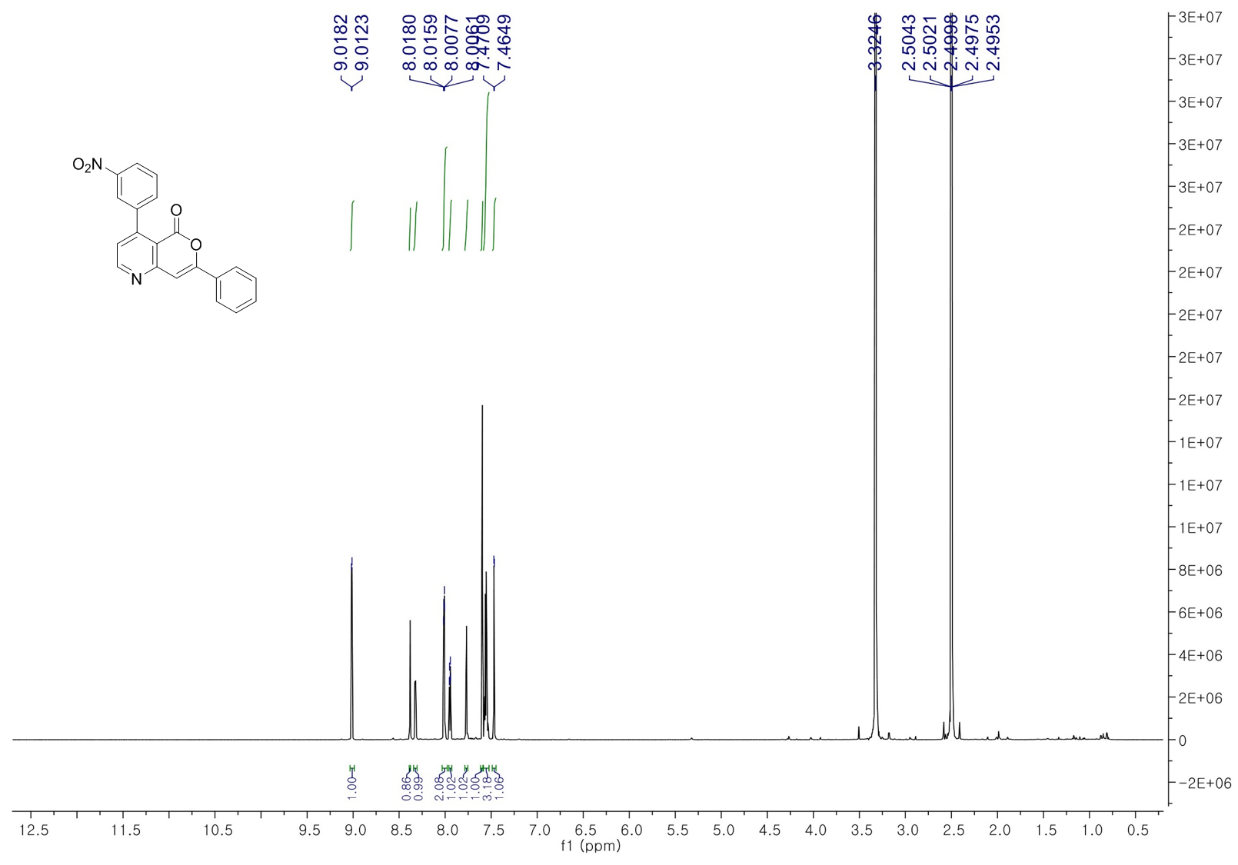 |
| ^13^C-NMR Spectra of **4f** (200 MHz, DMSO-d_6_)  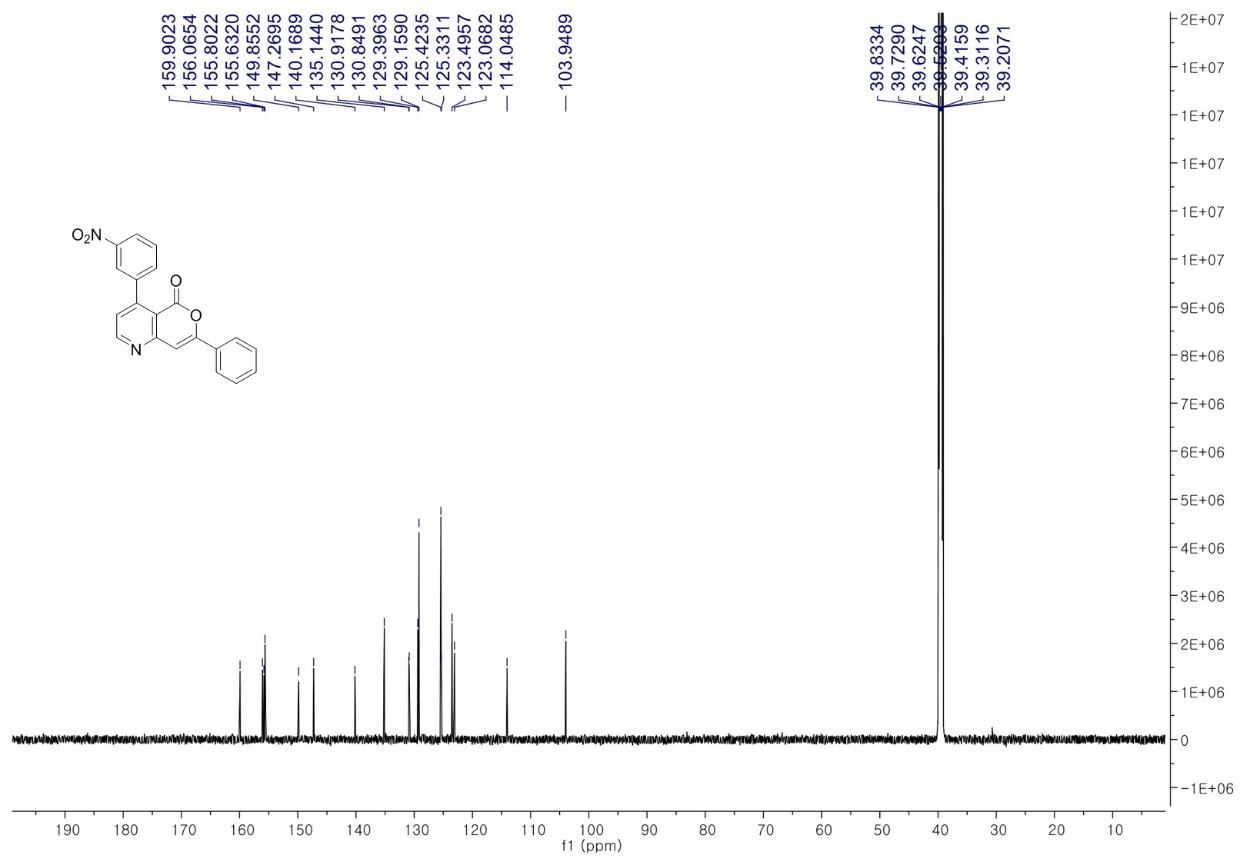 |

# References

Balducci, E., Bellucci, L., Petricci, E., Taddei, M., and Tafi, A. (2009). Microwave-assisted intramolecular Huisgen cycloaddition of azido alkynes derived from *α*-amino acids. *J. Org. Chem.* 74, 1314-1321. doi: 10.1021/jo802463r

Cui, X.-Y., Ge, Y., Tan, S. M., Jiang, H., Tan, D., Lu, Y., et al. (2018). (Guanidine)copper complex-catalyzed enantioselective dynamic kinetic allylic alkynylation under biphasic condition. *J. Am. Chem. Soc.* 140, 8448-8455. doi: 10.1021/jacs.7b12806

Mons, E., Jansen, I. D., Loboda, J., van Doodewaerd, B. R., Hermans, J., Verdoes, M., et al. (2019). The alkyne moiety as a latent electrophile in irreversible covalent small molecule inhibitors of cathepsin K. *J. Am. Chem. Soc.* 141, 3507-3514. doi: 10.1021/jacs.8b11027

Nishizawa, R., Nishiyama, T., Hisaichi, K., Hirai, K., Habashita, H., Takaoka, Y., et al. (2010). Discovery of orally available spirodiketopiperazine-based CCR5 antagonist. *Bioorg. Med. Chem.* 18, 5208-5223. doi: 10.1016/j.bmc.2010.05.057

Sakthivel, S., Sharma, A., and Balamurugan, R. (2017). Silver‐catalyzed synthesis of substituted pyridine derivatives from *N*‐propargylic *α*‐enamino esters. *Eur. J. Org. Chem.* 3941–3946. doi: 10.1002/ejoc.201700559

Prasad, J. V., Para, K. S., Tummino, P. J., Ferguson, D., McQuade, T. J., Lumney, E. A., et al. (1995). Nonpeptidic potent HIV-1 protease inhibitors:(4-hydroxy-6-phenyl-2-oxo-2*H*-pyran-3-yl) thiomethanes that span P_1_-P_2_' subsites in a unique mode of binding. *J. Med. Chem.* 38, 898–905. doi: 10.1021/jm00006a007

Uredi, D., Motati, D.R., and Watkins, E.B. (2019). A simple, tandem approach to the construction of pyridine derivatives under metal-free conditions: A one-step synthesis of the monoterpene natural product, (−)-actinidine. *Chem. Commun.* 55, 3270-3273. doi: 10.1039/c9cc01097a

Uredi, D., Motati, D.R., and Watkins, E.B. (2018). A unified strategy for the synthesis of *β*-carbolines, *γ*-carbolines, and other fused azaheteroaromatics under mild, metal-free conditions. *Org. Lett.* 20, 6336-6339. doi: 10.1021/acs.orglett.8b02441

Valverde, I. E., Delmas, A. F., and Aucagne, V. (2009). Click à la carte: Robust semi-orthogonal alkyne protecting groups for multiple successive azide/alkyne cycloadditions. *Tetrahedron* 65, 7597-7602. doi: 10.1016/j.tet.2009.06.093
